# Current Insights in Fungal Importance—A Comprehensive Review

**DOI:** 10.3390/microorganisms11061384

**Published:** 2023-05-24

**Authors:** Viorica Maria Corbu, Irina Gheorghe-Barbu, Andreea Ștefania Dumbravă, Corneliu Ovidiu Vrâncianu, Tatiana Eugenia Șesan

**Affiliations:** 1 Genetics Department, Faculty of Biology, University of Bucharest, 060101 Bucharest, Romania; viorica.corbu@yahoo.com; 2 Research Institute of the University of Bucharest–ICUB, 91-95 Spl. Independentei, 050095 Bucharest, Romania; ovidiu.vrancianu@yahoo.com; 3 Department of Microbiology and Immunology, Faculty of Biology, University of Bucharest, 060101 Bucharest, Romania; andreeadum29@gmail.com (A.Ș.D.); tatianasesan@yahoo.com (T.E.Ș.); 4 Academy of Agricultural Sciences and Forestry, 61 Bd. Mărăşti, District 1, 011464 Bucharest, Romania

**Keywords:** biodeterioration, filamentous fungi, fungal biotechnology, natural products, secondary metabolites

## Abstract

Besides plants and animals, the Fungi kingdom describes several species characterized by various forms and applications. They can be found in all habitats and play an essential role in the excellent functioning of the ecosystem, for example, as decomposers of plant material for the cycling of carbon and nutrients or as symbionts of plants. Furthermore, fungi have been used in many sectors for centuries, from producing food, beverages, and medications. Recently, they have gained significant recognition for protecting the environment, agriculture, and several industrial applications. The current article intends to review the beneficial roles of fungi used for a vast range of applications, such as the production of several enzymes and pigments, applications regarding food and pharmaceutical industries, the environment, and research domains, as well as the negative impacts of fungi (secondary metabolites production, etiological agents of diseases in plants, animals, and humans, as well as deteriogenic agents).

## 1. Introduction

The fungal kingdom comprises a plethora of eukaryotic species that proliferate in diverse environments; fungi also have essential roles as components of the microbiota, where they act as symbionts, endophytes, parasites, or saprotrophs [1,2]. Studies aiming to characterize the microbiota of diverse species across kingdoms have revealed an unexpected double nature of the fungi in the microbiome: they colonize higher eukaryotes from plants to humans [3]. In the meantime, as with all other eukaryotes, fungi host their microbiota, consisting of microbial communities that adhere to the hyphal surface, develop among the pseudotissues produced by hyphal aggregation, or colonize the fungal cytoplasm. Fungi, generally, can be microscopic to macroscopic, and include unicellular organisms such as yeasts and multicellular organisms such as filamentous fungi. Filamentous fungi grow long 2–10 μm thin filaments (hyphae) into intricate network structures (mycelium) that are observable to the naked eye and can grow to the centimeter to meter scale [4,5].

The filamentous growth mode and the secretion capacity of proteins and primary and secondary metabolites facilitate fungal proliferation in nature. Industry uses these properties to produce proteins, small molecule compounds, and, recently, mycelium materials. These bio-based products could be used as thermal and acoustic insulation and packaging [6]. Pure fungal materials are the result of complete substrate degradation or are obtained by removing the fungal skin from the surface of a substrate. The properties of the mycelium depend on the substrate, the type of fungus, and growth conditions [7].

Fungi could contribute to the aspiration to develop more sustainable manufacturing to protect the environment, being an optimal candidate to produce several products such as textiles and myco-leather, biofuels, building materials, wastewater treatment, and sustainable meat substitutes [8,9]. Besides industrial and food applications, fungi are also used in medicine for the production of a lot of compounds, e.g., antibiotics (such as penicillin), or a compound named cyclosporine, produced by *Tolypocladium inflatum*, as well as lovastatin, a drug for lowering the cholesterol from blood produced from *Aspergillus terreus* [10,11].

In agriculture, fungi play a significant role, including plant growth and protection. For example, mycorrhizal fungi establish a mutualistic association with plant roots, improving the plant’s nutrient uptake by increasing the surface area of the root system. This relationship helps the plant to access nutrients, such as phosphorus and nitrogen, that are not readily available in the soil [12,13,14]. Another essential agricultural fungal species is the endophytic fungi that colonize plant tissue. The complex interaction between endophytic fungi and plant tissue involves modulating the plant’s defense mechanism in terms of inhibiting phytopathogens and stimulating the growth of the plants even under biotic and abiotic stress conditions [15]. However, in a specific situation, some fungal species exert a less beneficial action on plant health, causing various plant diseases by colonizing roots, leaves, and tissue [16].

Regarding the environmental issues related to pollution and toxic wastes, fungi are quickly surfacing as essential protagonists, involved in practices such as the bioremediation of pharmaceutical compounds, agricultural wastes, or degradation of various pollutants.

Although fungi are widely spread in the environment and co-exist with many organisms, in certain circumstances, fungi, like any other microorganisms, can harm their host. Fungi are characterized by high resilience in stressful conditions and a remarkable ability to adapt to different environments. Infectious fungi can spread through the air and water and be transmitted by different carriers such as animals, humans, or plants [17,18]. Regarding the interaction of fungi with plant organisms, more than 80% of the plants from our planet are symbiotic with fungi. However, some of the fungal strains can sometimes enter inside plants through damaged leaves and stomata, thus turning themselves into plant pathogens with a high impact on plant health.

This review aims to characterize the beneficial roles of fungi used for a wide range of applications, such as the production of several enzymes (cellulase, proteinases, amylases, invertase, pectinase, chitinases, lipases, and, respectively, lignocellulolytic enzymes, citric acid, gluconic, itaconic, lactic, fumaric, malic, succinic, and oxalic acids); pigments (such as polyketide pigments, carotenoids, or melanin); applications regarding food (e.g., worldwide farmed mushrooms species such as *Agaricus bisporus*, *Pleurotus ostreatus*, *Flammulina*, and *Lentinula shiitake*, or the usage of filamentous fungi strains to produce different fermented foods such as cheese, bread, and beer, or the molds to manufacture fermented sausages, or alcoholic beverages production such as wine and beer); and in pharmaceutical industries, the environment (hydrocarbon degrading fungi, bioremediation, biofuels production, mycofiltration), and research domains (as research model organisms). Furthermore, this review summarize the negative impacts of fungi secondary metabolites production (B1, B2, G1, and G2 aflatoxins; A, B, and C ochratoxins; A, B, C, and P fumonisins; more than 200 trichothecenes; zearalenone and its derivatives; patulin), etiological agents of diseases in plants, animals and humans (e.g., *Alternaria* and *Cladosporium* genus comprise phytopathogenic species or can are responsible for human or animal infectious diseases), as well as deteriogenic agents of different substrates such as stone, wooden, paintings, textiles, parchments, paper and paper-based materials or heritage buildings.

In terms of novelty, this review brings together comprehensive data regarding the beneficial roles of fungi used for a wide range of applications (e.g., production of several enzymes, medicine, agriculture, industry, environmental safety, and research) and the damaging effects of fungi, acting as etiological agents of diseases in plants, animals, and humans. In addition, in this paper, we present the deterioration action of fungi on cultural heritage objects. Therefore, the rationale for choosing this subject is the impact of fungi on human health and environmental safety. People suffering from opportunistic and primary invasive fungal infections urgently need resources and research efforts to bring them new diagnostics and treatments regardless of commercial potential. In agriculture, the presented applications have the potential to improve crop yield, reduce the use of synthetic fertilizers and pesticides, avoid the use of toxic compounds, and promote sustainable agriculture practices. Thus, further attention must be paid to uncovering the biomolecules from fungi for agriculture and pharmaceutical applications through studying metagenomics, genomics, and proteomics.

## 2. Significance of Fungi in Different Sectors

### 2.1. Beneficial Roles

Fungi are the kingdom of those organisms whose species can populate practically all ecosystems. They are found as free-living and symbiotic unicellular or multicellular organisms and exist under varied morphologies [19]. They exist in almost all environmental types, from soil to water, and are best known for their essential roles in ecology as decomposers and symbionts. Fungi have also been used for centuries in several practices in the food and medicine fields. Recently, fungi have emerged as a valuable resource in modern biotechnology, with numerous applications across different sectors and as a sustainable candidate.

Fungi appear in various sizes, starting with microfungi such as molds and yeasts and progressing to macromyctes such as mushrooms or truffles. The macro-sized fungi are most often used for human consumption as supplements or food; on the other hand, the micro-sized fungi, including species such as *Aspergillus*, *Penicillium*, and *Saccharomyces*, are used for synthesizing enzymes and metabolites. According to these described abilities, fungi are considered one of the cornerstones of modern biotechnology [20].

Fungi indubitably dominate the biotechnology sphere; therefore, it is expected that their utilization is going to grow exponentially hereafter. They play a crucial role in various industrial processes, including manufacturing enzymes, pigments, vitamins, and so on [21]. Moreover, they are used to manufacture different types of food pigments such as benzoquinone (*Penicillium europium*), anthraquinones (*Paecilomyces farinosus*), melanin (*Aspergillus* spp.), and β-carotene (*Blackeslea trispora*) [8,9].

Fungi could contribute to the aspiration to develop more sustainable manufacturing to protect the environment, being an optimal candidate to produce several products such as textiles and myco-leather, biofuels, building materials, wastewater treatment, and sustainable meat substitutes [4,22,23].

Before we identifed the different fungi species and understood the definition of fungi, they had been used to produce various food in different parts of the world, such as fermented food, bread, wine, and cheese [20]. Today, a significant part of worldwide cuisine is represented by products made with fungi, usually products that result after fermentation.

#### 2.1.1. Medicine/Health

Natural products (NPs) produced by fungi are responsible for several effects, such as antimicrobial, immunosuppressive, anticancer, antidiabetic, immunomodulatory, and anti-inflammatory effects, many of which have been developed as treatments and have potential therapeutic applications for human diseases.

NPs such as non-ribosomal peptides (i.e., penicillin produced by *Penicillium rubens*; cephalosporin C by *Acremonium chrysogenum*, pneumocandins by *Glarea lozoyensis* and *Pezicula* (*Cryptosporiopsis*), or ribosomal peptides (amatoxins, piperazines), polyketides, lipopeptides, lipodepsipeptides and secondary metabolites produced by fungi mediate antimicrobial resistance and virulence and act in competition against other microorganisms [24].

Regarding antibiotic production by filamentous fungi, it has been proved that they initiated the golden era of natural antibiotics in the 20th century, as a consequence of extensive antibiotic use, especially in hospital settings, with the appearance of antimicrobial resistance phenomenon in the 21st century, especially to ESKAPE pathogens (*Enterococcus faecium*, *Staphylococcus aureus*, *Klebsiella pneumoniae*, *Acinetobacter baumannii*, *Pseudomonas aeruginosa*, and *Enterobacter* species) [25].

In Table 1, we list the NPs known as antimicrobial agents produced by different filamentous fungi strains or macromycetes.

#### 2.1.2. Fungi in Agriculture

Fungi can also act as biological control agents against plant pathogens. *Trichoderma*, for instance, demonstrated antagonistic effects against a wide range of plant-pathogenic fungi [13,70,71,72,73]. *Trichoderma* species have been used intensively in different biotechnological fields. Even so, they represent an outstanding contribution to agriculture because they show an excellent potential to defend against disease crops and attenuate the unfavorable conditions that can affect plant growth and stimulate plant growth [74,75,76,77,78]. These fungi are involved in biocontrol applications, versus fungi that can be plant-pathogenic, oomycetes, or nematodes [79]. Several fungi are used to control insect pests; for instance, species such as *Beauveria bassiana* attacks corn borer, *Verticillium lecanii* is known to control whitefly and aphids, and *Metarhizium anisopliae* are used against scarab larvae [34,80,81]. The action of the fungi is to infect the body surface, which leads to the attachment of the fungus to the integuments of the affected insect, where it will develop and continue to proliferate until the fungus entirely covers the insect [82]. Plant-parasitic nematodes depict another threat to the wellness of plants. Using fungi can be a sustainable strategy to avoid the intensive use of chemical nematicides. The leading group of filamentous fungi that were studied for the biocontrol of nematode pests is known as *Trichoderma.* The mechanisms involved in damaging nematodes can be found in the antibiosis (production of secondary metabolites), enzymes, or space competition [83].

The presented applications have the potential to improve crop yield, reduce the use of synthetic fertilizers and pesticides, avoid the use of toxic compounds, and promote sustainable agriculture practices. Therefore, fungi have an essential contribution to the agriculture sector, and they must be exploited [84,85,86].

#### 2.1.3. Industry

Fungi play an essential role in the food industry. They have been used since ancient times for various purposes, such as fermentation, production of enzymes, and as a source of food.

For centuries, humans learned to select and collect several macroscopic fungi known as mushrooms. They can be wild-harvested or commercially raised, and they are rich in protein; also, they can develop on inexpensive substrates, sometimes even agro-industrial wastes [87]. The most frequently farmed species worldwide are *A. *bisporus**, P. ostreatus, Flammulina, and Lentinula edodes, used in salads, soups, and other recipes. Many additional mushrooms are collected from the wild for personal use or sale, amongst which are king boletes, milk mushrooms, morels, chanterelles, truffles, black trumpets, and porcini mushrooms. They frequently appear in upscale cuisine [88].

Filamentous fungi have long been used to produce fermented foods such as cheese, bread, and beer. Soy sauce, miso, tempeh, mold-cheeses, and alcoholic beverages, including beer, wine, and spirits, are all products of traditional fungi and yeast fermentation techniques. Using fungi in these industries has significantly improved the final products’ quality, taste, and shelf life. One example of the use of filamentous fungi is cheese production. The first evidence of various cheese types dates from 897 for Gorgonzola, 1070 for Roquefort, and 1791 for Camembert [89]. Mold-ripening is the central aspect for an increased quality of the cheese for a reason during this process, as the flavor and consistency are improved. *P. roqueforti* makes blue cheese (Roquefort, Gorgonzola, Danish blue, etc.). It produces blue-green veins and gives the cheese its distinctive flavor after inoculation and growth [90]. On the other hand, *Penicillium camemberti* is inoculated on the surface of the cheese, and it changes the consistency—a soft texture—more than the flavor. These types of cheese are Camembert and Brie [91].

Molds have been used to manufacture fermented sausages since the Greek and Roman empires, where fermented and air-dried sausage had widespread popularity among peasants due to its long-term stability at room temperature as a replacement for refrigerators nowadays. Various types of meat, including cow, goat, horse, lamb, pork, and chicken, are used to make salami [92]. *Penicillium nalgiovense* is most frequently used as a starting culture when curing fermented meat products; also, the species is recognized as safe to use [93].

The potential use of fungi is great because, currently, farming is conducted at higher rates. There is a need to find other alternatives to avoid soil erosion speeding up and environmental pollution; therefore, the alternative is microorganism cultivation aiming at the production of edible biomass [94]. Quorn™ is a company with fungi-based food products producing dried fungal biomass from *Fusarium venenatum*. The product is a mycoprotein, low-fat, low-calorie, cholesterol-free food that is highly popular in Anglo-Saxon nations [95]. Mycoprotein is known to help against body weight issues because it gives a feeling of satiety. Therefore, an investigation of the safety of *F. venenatum* mycelia for human eating was conducted between 1970 and 1980 following the isolation and subsequent suitability testing of *F. venenatum* (at that time, still known as *F. graminearum*). The potential for the fungus to create mycotoxins was a significant worry, as many *Fusarium* species are notorious for producing these toxins due to their role as phytopathogens [77,96].

For the production of Japanese traditional goods, such as seasonings or alcoholic drinks (soy sauce, miso, soju), *Aspergillus oryzae* is the predominant species used in fermentation processes. Starting in China between 3000 and 2000 years ago, koji became a popular item in Japan with the purpose of a starter for secondary fermentation [97]. Applying *A. oryzae* spores to heated rice produces koji. The resulting mixture is combined with soybeans or other steamed rice, water, and yeasts to ferment. Besides *A. oryzae*, other filamentous fungi are used to make koji, such as *Aspergillus sojae*, *Aspergillus kawachii*, and *Aspergillus awamori* [98].

Manufacturing alcoholic beverages such as wine and beer is another common usage of the fungal species in the food industry. For example, *Botrytis cinerea*, a plant-pathogenic fungi, is exploited in southern France and other locations to increase the sugar level in grapes before harvesting, producing “noble rot,” a sweet and premium wine [99]. For centuries, mankind has used *Saccharomyces* yeasts to make beer, bread, and wine. Since high quantities of ethanol harm most other bacteria, yeasts have historically been utilized as efficient methods to protect food and beverages’ nutritional value and security. The most well-known among all the beneficial yeasts is *S. cerevisiae*. It can be utilized to make wine, beer, and bread. Additionally, kefir is created by the symbiotic relationship between bacteria and *Saccharomyces* yeasts [100].

One of the numerous beneficial features of fungi is the synthesis of fungal enzymes and organic acids. Cellulase (*Penicillium funiculosum*, *Trichoderma viride*), α-amylases and invertase (*A. niger*, *A. oryzae*), proteinases (*A. oryzae*), and citric acid (*A. niger*) can be listed among them. 

Several fungal species have been used regarding food processes since the beginning of agriculture. Fungal biodiversity is undoubtedly an important provider of resources for food as well as other higher value uses.

In the present, society confronts many manufactured challenges, among which high pollution levels or the lack of nutritional resources to support population growth occupy a central position [101,102]. The transition to a zero-carbon sustainable bioeconomy is the only direction that offers humankind the possibility to address these challenges, and it involves the transformation of a linear economy into a sustainable circular economy. Microorganisms are critical players in the circular economy since they harbor many intrinsic characteristics that recommend them for biobased industrial applications [23,102]. Among microbial strains with industrial potential and a high impact on the circular bio-economy, fungal strains have the unique metabolic ability to convert many organic materials (including wastes) into various by-products relevant to different industrial applications [23].

##### Metabolites Produced by Fungi with Industrial Applications

Different chemical compounds with industrial applications, such as organic acids, enzymes, flavors, vitamins, and colorants, might be obtained more cost-efficiently using fermentation processes based on fungal strains [103].

Organic acids are used in the food industry mainly as acidulants, flavoring agents, or preservatives, but their biotechnological potential is not limited only to the food industry. Citric acid is a weak organic acid highly demanded worldwide, especially in the food and pharmaceutical industries. The bioproduction of citric acid comprises the traditional process of industrial-scale fermentation. Among the most common organic acids used in industry is citric acid, which has a variety of uses, and the global market for 2025 is estimated at USD 3.6 billion [104]. Besides the use in food and beverage industries, the mentioned organic acid is utilized in pharmaceutical, cosmetic, and detergent industries [104]. Karl William Scheele is recognized for the isolation of citric acid for the first time from lemon juice in 1784. James Currie discovered in 1917 that *A. niger* could create citric acid from sugar using the surface fermentation method, which later served as the foundation for *Aspergillus*’s application in industrial production. Because of its higher production yield, *A. niger* is better than other microorganisms for the industrial synthesis of citric acid. It provides excellent yields, can ferment a variety of inexpensive basic materials, and is simple to handle [105].

In the food and beverage industry, it is used as a preservative or as a flavors and aromas enhancer, for preventing the deterioration of frozen food products or for the development of non-toxic plastic films for foodstuffs protection, and also as an emulsifying agent for ice cream and cheese-based food products [106]. In the pharmaceutical industry, as an antioxidant, citric acid is used to preserve vitamins, as a pH buffer, and in association with other chemicals such as iron (as iron citrate tables) or for the development of citrate-based biomaterials with potential use for regenerative engineering [107,108]. Other possible applications of citric acid are for the chemical industry, mainly for producing conditioners and laundry detergents or as chelating agents in cleaning solutions used for removing limescale [106,109,110]. Every year more than 6 million tons of citric acid are used for beverages, food, detergents, cosmetics, and pharmaceutical production. High quantities of citric acid are assured using the fungal submerged fermentation of sucrose and molasses or synthetically from acetone or glycerol. Numerous *Aspergillus* strains belonging to *A. niger*, *A. awamori*, *Aspergillus foetidus*, *Aspergillus wentii*, *Aspergillus aculeatus*, *Aspergillus carbonarius* species or *T. viride*, *Penicillium restrictum*, and *Mucor piriformis* are currently evaluated as being good producers of citric acid [103,105,111]. However, among them, *A. niger* is considered most suitable for industrial production due to its high ability to assimilate and ferment many cheap agro-industrial derived materials, and thus it is a cost-effective technology [105]. Among cheap agro-industrial wastes used for citric acid production using *A. niger*, it is worth mentioning pineapple peels [112]; apple processing wastes [113]; banana peels [114]; cocoa pod and coffee husk processing wastes [115,116] or sweet potato starch hydrolyzate [117]. Gluconic acid is another highly valuable organic acid for the food industry, which can be obtained using fungal fermentation [103]. Gluconic acid is a mild organic acid frequently used to pickle foods, prevent milkstone in the dairy production industry, or for clean cans used in these circumstances. Gluconic acid derivatives such as D-glucono-δ-lactone are important as leavening agents for preleavened products, for reducing fat absorption in doughnuts, for the coagulation of soybean proteins in tofu manufacture, and for improving the heat stability of milk [118,119]. Gluconic acid salts (sodium gluconate, calcium gluconate, ferric gluconate) have a broadened industrial potential from metallurgy where they can act as alkaline derusting agents and anticorrosive agents to use as additives to cement [120,121]. Calcium and iron salts of gluconic acid have important biomedical potential, being used for calcium therapy for osteoarthritis [122] and the treatment of anaemia [123], and they also can be used in animal feed [124] or in agriculture as foliar feed formulations [118]. Annually, more than 60 thousand tons of gluconic acid and its derivatives are produced worldwide through various chemical, electrochemical, or fermentation processes. By far, fermentation is considered the most efficient and dominant technique that can be used to obtain gluconic acid. Among the microbial strains involved in gluconic acid production through fermentation, *A. niger*, *Penicillium funiculosum*, *Penicillium variable*, *Penicillium amagasakiens*, and members of *Glicladium*, *Scopulariopsis*, *Gonatobotrys*, and *Endomycopsis* genera are well known [118]. *A. niger* is considered a main fungal species that can produce gluconic acid at an industrial level. Members of this species produce all the enzymes involved in converting carbohydrates, such as glucose, into gluconic acid. Since industrial production requires the reduction of costs, a strategy presented in the scientific literature was to replace the substrate represented by glucose with cheaper raw materials such as breadfruit hydrolysate [125], grape must [126], waste office paper hydrolysate [127], whey [128], dry dilute acid pre-treated corn stover [129], corn starch [130], sugarcane molasses [131], or banana must [119].

Apart from citric and gluconic acids, fungi are also used for obtaining other organic acids such as itaconic, lactic, fumaric, malic, succinic, and oxalic acids, but to a lesser extent [103]. Itaconic acid has been commercially available since the mid-twentieth century, significant for the industrial production of adhesives, detergents, and shampoo formulations. More than that, its vinyl esters are relevant for producing plastics, elastomers, and coatings with light colors for carpets and book covers [132]. For the biomedical field, itaconic acid is used for ophthalmic, dental and drug delivery fields [133,134]. *A. terreus* and *Ustilago maydis* species are considered model organisms for producing itaconic acid. They are also characterized by outstanding tolerance to acidic pH values. However, apart from them, other fungal species such as *Ustilago vetiveriae*, *Ustilago xerochloae*, or *Aspergillus niveus* were described as being able to produce this organic acid. As an alternative growth substrate for the production of itaconic acid using fungi, it is worth mentioning enzymatically digested wood chips [135], corn stover hydrolysate [136], pre-treated rice husk [137], sweet potatoes, wheat flour, corn starch [138] beech wood [139,140], and glycerol [141]. Lactic acid is widely used in the food industry as a preservative (preventing the proliferation of spoilage microorganisms) and for the production of yogurt and cheese (involved in decreasing pH and casein aggregation) [142]. Apart from the food industry, lactic acid is a precursor for propylene glycol and acrylic polymers that can be used to develop biodegradable packaging and labeling materials [143,144] and also for biomedical prosthetic devices or sutures [145]. Although it can be obtained through chemical synthesis, the main advantage of fermentation processes for obtaining lactic acid for the food industry is that selecting the right microbial strain can yield a pure form of L(+)-lactic acid, which is preferred since it is not harmful to humans [146]. Usually, lactic acid bacteria are preferred for lactic acid production, but since this group of microorganisms exhibits special nutritional requirements, fungal strains represent a cheaper yet productive alternative. *Rhizopus* spp. represents the most critical fungal genera that can be used for lactic acid production. Members of this genus have the advantage of producing only L-lactic acid isomers, thus reducing the cost associated with the purification of the fermentation products. Although glucose is preferred as a carbon source for producing lactic acid using *Rhizopus* strains, other substrates such as raw starch from potatoes, cassava, wheat, corn, and rice wastes [147,148,149,150] or lignocellulose wastes [151,152,153] can contribute to cost reduction [146]. Malic and fumaric acids are other valuable organic acids that can be obtained using fungal strains. Their industrial applications vary from the manufacture of chemical products such as resins, biodegradable polymers, lubricating oils, inks, plasticizers, or lacquers to the production of food and pharmaceutical additives (acidulants, flavor enhancers, precursors for malic or aspartic acid) or drugs (including those with antimicrobial properties, antioxidant, and anticarcinogenic effects) [154]. Fungi species such as *Rhizopus arrhizus*, *Rhizopus oryzae*, *Mucor* spp., *Cunninghamella* spp., or *Aspergillus* spp. are presented as good producers of fumaric acid, both through aerobic and anaerobic fermentation, while for malic acid good results are reported for *A. oryzae*, *A. niger* and *Aureobasidium pullulans* [155,156,157]. Good yields of fumaric acid production were obtained using different substrates for fungal biomass accumulation, of which it is worth mentioning: apple industry waste biomass [158] or different food wastes disposed by restaurant, kitchens, and cafeterias [155,159,160].

Filamentous fungi are also used for producing enzymes at large scales. Their versatile metabolism assures obtaining large quantities of amylases, proteases, pectic enzymes, galactosidases, lipases, chitinases, or lignocellulolytic enzymes.

Amylases with biotechnological importance are extracellular enzymes involved in starch degradation. These enzymes represent approximately 25% of the world enzyme market, relevant to many industrial processes such as those in the food, fermentation, textile, paper, and pharmaceutical industries (Table 2). Three types of amylases are produced using microbial strains: α-amylases, β-amylases, and γ-amylases. α-amylases or endo-1,4-α-D-glucan glucohydrolase (EC 3.2.1.1) catalyze the hydrolysis of random 1,4-α-D-glycosidic bonds between glucose units from short linear amylase chains [161]. Unlike these, the β-amylases or β-1,4-glucan maltohydrolase (EC 3.2.1.2) are responsible for the hydrolysis of the second 1,4-α-D-glycosidic bond from the non-reducing end of the starch molecule, thus producing disaccharides such as maltose.

On the other hand, γ-amylases and glucan 1,4-α-glucosidase (EC 3.2.1.3) are usually highly stable enzymes in acidic conditions, and these enzymes are mainly responsible for the cleavage of 1,4-α or 1,6-α-D-glycosidic bonds on the external glucose residues of amylose or amylopectin from the non-reducing end, thus producing only glucose [162]. The industrial production of amylases usually involves submerged fermentation, but recently solid-state fermentation has received greater interest due to its superior productivity, reduced energy requirement, and simpler fermentation media. In addition, many studies have reported the optimal production conditions of fungal amylases in terms of the cultivation conditions (pH, presence of different inhibitors, temperature a.s.o) and the substrate used to obtain the biomass (Table 2).

Proteases constitute a large group of enzymes responsible for hydrolysis peptide bonds. In general terms, according to the position of the cleaved peptide bond, proteases can be divided into two major groups: endopeptidase and exopeptidase. Fungal strains can produce both types of protease, thus having great importance for their production. Fungal proteases can be obtained using both submerged fermentation and solid-state fermentation. Both options seem more advantageous in fungi than other protease-producing organisms (microbial or not) [103,163]. Among the protease-producing fungi, thermophilic fungi such as *Thermoascus aurantiacus* [164] or *Thermomyces lanuginosus* [165] are of great interest since they possess the ability to secrete thermostable proteases that act in the temperature range 60–85 °C.

Pectinase, in general terms, refers to a group of enzymes that catalyzes pectic substance depolymerization (pectin hydrolases and lyases) and de-esterification (pectin esterases). Pectinase represents approximately 10% of the overall production of enzymes, and its utilization is highly valuable for the food industry, but not only. Microbial pectinases, in general, are relevant for the natural carbon cycle involved in the decomposition of dead plant material. However, for the producing microorganism itself, these enzymes represent a tool in the phytopathologic process and plant-microbe symbiosis [166]. Either way, pectinase can be successfully used in the industry for various applications (Table 2).

According to their substrate specificity, the galactosidases are glycoside hydrolases classified as α-galactosidases or β-galactosidases. α-Galactosidases (EC 3.2.1.22) catalyze the removal of α-linked terminal non-reducing galactose residues from small oligosaccharides. This enzyme is also responsible for the cleavage of α-1,6 linkage between galactose and glucose in melibiose. α-Galactosidases have proven helpful in the food and feed industry, mainly increasing the sucrose yield by eliminating raffinose [167]. β-Galactosidase (EC. 3.2.1.23), also known as lactase, is responsible for the hydrolysis of D-galactosyl residues from polymers. Fungal β-galactosidases are highly stable to acidic pH, thus being an excellent instrument for whey reintegration into the economic circuit [168,169,170].

Chitinases (EC. 3.2.1.14) are enzymes responsible for chitin—the second-most-abundant polymer found in nature—degradation. Fungi are the most important group of microorganisms able to produce and secrete chitinase. Fungal chitinases are the only enzymes that can efficiently degrade chitin by hydrolyzing chitin to form chito-oligosaccharides with a minimum chain length of two carbon atoms [171]. Apart from their involvement in fungal morphogenesis, cell division, mycoparasitism, and autolysis, from a biotechnological point of view, chitinases are essential for the functional reintegration of chitin trapped in the biomass in the economic circuit [172].

Lipases (EC. 3.1.1.3), or triacylglycerol hydrolases, catalyze glycerol and fatty acids hydrolysis. It was also noticed that their processes, including the extraction and purification of lipases from fungi, are comparatively more accessible and cheaper than other sources of lipases. Fungal lipases have applications not only in the hydrolysis of fats and oils (triglycerides) but are also involved in synthetic reactions such as esterification, acidolysis, alcoholysis, interesterification, and aminolysis. Although some fungal species produce intracellular lipases, most can secrete this enzyme outside the cell. Major genera of filamentous fungi capable of producing lipases are *Rhizopus*, *Aspergillus*, *Penicillium*, *Mucor*, and *Geotrichum*, and the lipases produced have special biotechnological applications that are intensively studied both from a functional and genetic point of view [173,174].

Lignocellulolytic enzymes are involved in lignocellulose degradation, and this group of enzymes includes ligninases, hemicellulases, and cellulases. Ligninases are responsible for deleting lignin into more minor compounds that microorganisms can assimilate. In general, ligninases can be divided into laccase or phenol oxidase and peroxidases or lignin peroxidases. Laccases (EC 1.10.3.2), or *p*-diphenol: dioxygen oxidoreductases, are the enzymes responsible for the attack of the phenolic subunits of lignin leading to Cα-Cβ cleavage and aryl-alkyl cleavage [175]. Lignin peroxidase (EC. 1.11.1.14), or diaryl propane oxygenase, is a heme-containing enzyme that catalyzes lignin’s hydrogen peroxide-dependent oxidative degradation. These enzymes belong to the oxidase group and are mainly used for reducing environmental pollution. Ligninases are widely found in nature and produced by various plants, fungal species, or bacteria. Among fungal species, white rot fungi are the best ligninases producers, and their biotechnological and industrial potential has been intensely studied in recent decades [176]. Cellulases are a group of enzymes that contain endoglucanase (EC 3.2.1.4), exoglucanase (EC 3.2.1.91), and β-D-glucoside glucanhydrolase (EC 3.2.1.21). Among the microorganisms, fungi are the principal cellulose decomposers, responsible for about 80% of the cellulose breakdown on earth. In industry, fungal cellulases are usually preferred, being much easier to be obtained in large quantities. Fungal species, including *T. reesei*, *Rasamsonia emersonii*, *Aspergillus* spp., and *Penicillium* spp. produce extracellular cellulases during their growth in aerobic conditions, and thus are promising candidates for various industrial applications [177,178].

**Table 2:** The most common filamentous fungi enzyme producers and their applications in industry.

Enzymes			Fungal Species	Non-Conventional Growth Substrates	Applications	References
Amylases			*A. niger* *A. oryzae* *A. fumigatus* *Aspergillus flavus*; *A. awamori*; *A. kawachii*; *Penicillium brunneum*; *Penicillium expansum*; *P. roqueforti*; *P. camemberti*; *Helminthosporium oxysporum*; *Penicillium frequestans* *P. chrysogenum* *Penicillium fellutanum*	Coconut oil cake; groundnut oil cake; sesame oil cake; olive oil cake; wheat bran; corncob leaf; rye straw; wheat straw; banana waste; residues obtained from rice husking; cassava peels; yam peels; pomegranate peel; molasses	in bread-making it is used to break down complex sugars found in flour in order to make the bread-making process faster and to improve the texture and volume of the final product; increases the shelf life of bread and bread derivatives by functioning as anti-staling agent; for the production of glucose fructose syrup, which can be used in the beverage industry as artificial sweetener; can be used animal feed pre-treatment, improving the fiber digestibility; as additive for clothing and dishwasher cold-water detergents, being stable at low temperatures and high pH values; in the textile industry it can be used for the production of fabrics, particularly for the removal of starch, which is used as a strengthening agent; in the paper industry amylases can be used for the hydrolysis of high-molecular-weight starch, which is present in coated paper. It promotes the quality (smoothness and strength) and erasability of paper; for the production of fuel alcohol, amylases can be used for the conversion of starch into fermentable sugars.	[161,162,179,180,181,182,183,184,185,186]
Proteases			*A. flavus*; *Aspergillus ochraceus* *Conidiobolus coronatus*; *Rhizomucor miehei*; *Endothia parasitica*; *Mucor circinelloides*; *Mucor pusillus*; *P. camemberti*; *P. citrinum*; *Penicillium griseoroseum*; *Penicillium restrictum*; *P. roqueforti*; *A. flavus*; *A. oryzae*; *A. niger*; *R. oryzae*; *T. reesei*; *Trichoderma harzianum*	Wheat and rice bran, soybean meal; oil seed cake	in the food industry they are used for improving nutritional and functional value of food products by enhancing their digestibility, by reducing the amount of proteic allergenic compounds; altering the viscoelastic properties of dough; the improvement of the quality, mainly consistency, of protein-rich foods; beef meat tenderization; to reduce or to modify wheat gluten content; for soy sauce and soy-derivatives production improvement by lowering their bitterness and solubility. In the dairy industry, proteases are used in cheese manufacturing for macropeptides production; for dietetic and health products production using protein hydrolysates obtained from casein; in the detergent industry for improving the detergent formulations in order to enhance their ability to remove tough stains; in the leather industry they can be used as alternatives to chemical treatments needed for soaking, dehairing and bating of raw materials; in the pharmaceutical and cosmetic industry these enzymes can be used for keratin elimination in acne or psoriasis; for depilation; for improving ungual drug delivery; in biomedicine, they can be used for enhancing the scar healing process or for obtaining plasma hydrolysate with antioxidant properties.	[49,163,187,188,189,190,191,192,193,194,195]
Pectinase			*A. niger* *A. flavus* *A. sojae* *A. terreus* *Alternaria citri* *Claviceps purpurea* *Fusarium moniliforme* *Botrytis cinerea* *A. kawakii* *Thermoascus aurantiacus* *Acrophialophora nainiana* *Aspergillus japonicus*	Wheat bran; rice husk and bran; papaya peel; mango peel; sugarcane bagasse; sunflower head; grape and strawberry pomace	in the food industry they are used for apple and grape juice clarification, wine production (for increasing the juice yield by decreasing the viscosity), and coffee-beans processing to remove the mucilage coat; pre-treatment of wastewater from the processing of vegetable food too rich in pectin; the processing of textile fibers such as: flax, jute, hemp; degumming of jute, sunn hemp, flax, ramie and coconut fibers; biosourcing of raw cotton; the extraction of vegetable oils from coconut, palm, sunflower seed, rape seed olives and pre-treatment of paper pulp to remove acidic polysaccharides; for animal feed production to reduce the feed viscosity and to enhance nutrient absorption.	[166,196,197,198,199,200,201,202,203,204]
Galactosidases	α-Galactosidases		*Mortierella vinaceae* *Tricholoma matsutake* *A. niger* *A. oryzae* *A. fumigatus*	Soybean meal and wheat bran red gram plant waste; soy flour	processing of soya, sugar beet, and guar gum for the food industry; improving the nutritional value of soy milk by the degradation of indigestible oligosaccharides; animal feed processing to remove its high content of raffinose-family oligosaccharides, which are associated with their negative impact on intestinal health; for obtaining functional food ingredients such as prebiotic galacto-oligosaccharides; as digestive aids in humans for preventing digestive disorders such as flatulence, bloating or abdominal pain.	[101,102]
	β-galactosidase		*A. niger* *A oryzae* *A. flavus* *Aspergillus uvarum* *P. brevicompactum* *F. oxysporum*	Lemon peel, pineapple peel, musk melon peel, banana peel, musambi peel, pomegranate peel, orange peel; soybean residue, okara, soymilk; wheat straw, rice straw, and peanut pod	in the dairy industry they are used for preventing lactose crystallization in sweetened, condensed, and frozen dairy products or for developing lactose-free dairy products for lactose-intolerant humans; for developing prebiotics based on galacto-oligosaccharides to promote the growth and the establishment of bifidobacteria in the intestine.	[168,205,206,207,208,209]
Chitinases			*Thermomyces lanuginosus*; *T. viride*; *T. harzianum*; *A. nidulans*, *A. fumigatus*, *P. chrysogenum*	Wheat bran; rice bran; chitin flakes; waste products obtained from crabs, shrimps and prawn	biocontrol agent against *Eldana saccharina* and phytopatogenic fungi; for chitosan-oligosaccharides-based antimicrobial drug production or as anti-cervical cancer compounds.	[171,210,211,212,213,214]
Lipases			*Mucor circinelloides* *Penicillium aurantiogriseum* *Rhizopus rhizopodiformis* *Rhizomucor pusillus* *Rhizopus oligosporus* *P. restrictum* *Penicillium simplicissimum* *Aspergillus carneus* *Penicillium verrucossum* *P. chrysogenum* *A. awamori* *A. terreus* *Fusarium solani*	Soya bean oil; olive oil cake; babassu oil cake Almond meal; mustard oil cake, sunflower oil; soybean bran; rice bran oil; olive mill wastewater	flavor development in dairy products and processing of meat, vegetables, fruit, baked foods, milk products, and beer; development of functional foods and nutraceuticals, such as trans-fatty acid-free margarines; lipases immobilized on pH/oxygen electrodes along with glucose oxidase serve as lipid biosensors for the determination of triglycerides and blood cholesterol; for esterification reactions and aroma ester production; biodegradation of diesel oil hydrocarbons and bioremediation of polyaromatic hydrocarbons-contaminated soil; to assist in the removal of size lubricants in order to provide the fabric with better absorbency for enhanced levelness in dyeing; for improving hydrophilicity and anti-static ability of PET (polyethylene terephthalate) fabrics; as a biocatalyst in personal care products such as skin and sun-tan creams, bath oils or for hair waving preparation.	[215,216,217,218,219,220,221,222,223,224]
Lignocellulolytic enzymes	Cellulase		*A. niger* *T. reesei* *Aspergillus heteromorphus* *A. fumigatus* *R. oryzae*	Wheat straw and bran; maize straw; banana peel Coir waste; grass; sugarcane bagasse; corn cob residue	pulping and deinking of wastepaper; improving the softness and appearance of cellulose-based textiles; improving the dye absorbance of the fibers and to remove excess dye; textile waste hydrolysis for the recovery of glucose and polyester; increasing the aroma and the taste of citrus fruits; production of bioethanol by converting the cellulosic renewable resources into glucose; increase soil fertility by promoting the straw decomposition.	[225,226,227,228,229,230]
	Ligninanses	Laccases	*Aspergillus niveus* *Rhizoctonia solani* *B. cinerea* *Myceliophthora thermophila* *Pycnoporus cinnabarinus* *Trametes villosa* *Coriolopsis gallica* *Coprinopsis cinerea*	Wheat bran, rice husk, mango peel, orange peel, groundnut husk and saw dusk	improvement of breadmaking performances of oat flour and the textural quality of oat bread; prevent undesirable changes such as discoloration, clouding, haze, or flavor changes in beer, fruit juices, and wine, improving their shelf life by removing phenols such as coumaric acids, flavans, and anthocyanins; improve the brightness and strength properties of the pulp; internal sizing of paper; bioremediation of pollutants such as bisphenol, diclofenac; 17-α-ethinulestradiol from wastewater; detoxify agricultural byproducts including olive mill wastes or coffee pulp; bioremediation of pulp and paper industry waste by effecting direct dechlorination and the removal of chorophenols and chlorolignins from bleach effluents; indigo carmin, Congo red, aniline blue dyes decolorization; for the development of laccase-containing biosensors for detecting O_2_, glucose, aromatic amines, phenolic compounds, and a wide variety of reducing substrates.	[175,231,232,233,234,235,236,237,238]
		Peroxidases	*Phanerochaete chrysosporium*; *A. sclerotiorum* *Cladosporium cladosporioides* *M. racemosus* *Neurospora discreta*	Cocopeat, sugarcane bagasse	development of cosmeceutical and dermatological products used for skin-lightening products; decolorization of Congo red, Poly R-478 and Methyl green.	[239,240,241]

*Pigments*: Filamentous fungi are also known for their ability to produce natural pigments with a high potential of replacing artificial synthetic dyes. Even today, synthetic colorants are widely used in foodstuff, cosmetics, pharmaceutical, and textile manufacturing, but some are hazardous to human health. In this context, there is a strong interest in replacing these colorants with their natural alternative [242]. Fungal pigments are an excellent alternative to synthetic dyes, which are easy to obtain and less expensive [243]. Many fungal species of *Aspergillus*, *Fusarium*, *Penicillium*, and *Trichoderma* genera produce large amounts of pigments during their growth. Fungal pigments are classified into polyketides, polyketide-derivates, carotenoids, and melanins [242]. Polyketide pigments have a polyketide chain with four or eight C2 units, and in this group are included secondary metabolites such as anthraquinone, hydroxyanthraquinones, naphtoquinone, and azaphilone. These compounds are responsible for a wide range of colors from yellow to red and even blue shades [244] and are successfully used for textile dyeing and as antibacterial agents. Polyketide pigments-producing fungi mainly belong to *Fusarium sporotrichioides*, *Penicillium* spp., *Aspergillus ustus*, and *Monascus purpureus*, and their potential utilization in the benefit of humankind varies from developing anti-aging, anti-acne, and skin-whitening agents to anticancer drugs [245]. Some species of fungi are used in the production of pigments, such as *Monascus* species, which produce red, orange, and yellow pigments used as natural colorants in the food, cosmetic, and pharmaceutical industries, as well as the dyeing, textile, and printing domains [246]. Carotenoids are tetraterpenoid pigments comprising xanthophylls and carotenes. In this class of natural pigments, more than 750 chemical compounds are included; among them, most important is β-carotene, a vitamin A precursor and a vital antioxidant agent. It is used as an orange-red pigment in the food industry and is mainly produced by *Blakeslea trispora* strains [103]. Other carotenoid-producing fungi belong to *Aschersonia aleyrodis*; *Aspergillus giganteus*; *B. trispora*; *F. fujikuroi*; *M. circinelloides*; *B. trispora*; *Sclerotinia sclerotiorum*; *Fusarium sporotrichioides*; *Phycomyces blakesleeanus*; *Neurospora crassa*; *Puccinia distincta and Allomyces arbusculus* [247]. At the industrial level, *B. trispora* is widely known, with members of this species being used to produce β-carotene [245]. More than that, fungal carotenoids can be obtained in a cost-efficient manner using alternative growth substrates such as waste cooking oil [248], deproteinized hydrolyzed cheese waste [249], or oat flakes/spent malt grain [250]. Melanins are dark brown or black pigments widely found in animals, plants, and microorganisms. From an industrial point of view, fungal melanins have gained significant interest in the last decades, being eco-friendly and biodegradable. According to their chemical structure and precursors involved in their biosynthesis, fungal melanins are classified into five main groups: eumelanin, 1,8-dihydroxynaphtalene melanins, pyomelanin, pheomelanin, and glutaminylhydroxybenzene melanin [251]. Being extremely diverse from a chemical point of view, fungal melanins positively impact biomedicine; the dyeing industry; the food industry for developing new packaging materials; for cosmetic industry; and environmental protection a.s.o [252].

#### 2.1.4. Fungi and the Environment

Currently, concerns about environmental safety are emerging since there are issues relating to pollution and toxic wastes. Therefore, there is a need to develop new strategies to sustain the environment’s health. Fungi are an integral part of the environment and play essential roles in many ecosystems. Besides the impact on nutrient cycling, decomposition, and soil health, fungi exhibit great potential for developing strategies that enhance environmental protection. For instance, in the fight against pollution, climate change, and various other issues, fungi are quickly surfacing as essential protagonists, involved in practices such as the bioremediation of pharmaceutical compounds, agricultural wastes, or degradation of various pollutants.

Mycoremediation is mediated by two mechanism types: enzymatic (fungal secreted enzymes) and non-enzymatic (adsorption of toxic compounds inside the cell wall, biosurfactants production) [253]. For example, filamentous fungi belonging to the *Trichoderma*, *Penicillium*, and *Aspergillus* genera are able, through absorption mechanisms, to absorb heavy metals such as copper and cobalt [254].

A study by Asemoloye et al. established that two fungal strains belonging to *Mucor irregularis* and *A. oryzae*, isolated from oil-contaminated places, could be used to clean up the soil after hydrocarbon contamination. Moreover, the two fungi displayed a remarkable capacity to degrade hydrocarbons [255].

Filamentous fungi are highly efficient in the process of decolorization. There is evidence that filamentous fungi produce the enzymes laccase and manganese peroxidase to achieve this. By converting complex synthetic dye molecules into non-colored, safer, and environmentally secure structures, fungal laccases were widely used for bioremediation [253]. It has been demonstrated that microorganisms exploit agricultural waste, specifically cellulose—the most renewable source of biomass in the biosphere—to produce valuable goods, such as sugars, cheap energy resources, and enzymes. Waste products from industry and agriculture are some of the things that pollute the environment [228]. Their transformation into beneficial products might lessen the issues they create. These wastes, including grains, leaves, corn cobs, and other materials, are underutilized.

As mentioned in the previous section, fungi can secrete cellulase, enzymes responsible for breaking down the cellulose in agricultural wastes into simple glucose molecules. Cellulolytic fungi, including *Chaetomium*, *Fusarium*, *Myrothecium*, and *Trichoderma*, produce celluloses through cellulolysis [256].

Another way to save the environment is using renewable energy from living organisms, known as biofuels. Biodiesel production was greatly enhanced by cultivating filamentous oleaginous fungi with lignocellulosic biomass, such as *Mortierella isabellina* and *Aspergillus terreus* [257]. A second way to produce biofuels is to develop bio-ethanol. Biomass of crops from grains and corn, rich in sugar and starch, is the base of bio-ethanol production. Filamentous fungi can convert sugars to ethanol. The pre-treatment of the biomass is realized with the aid of fungi and lignin-degrading enzymes (pectinases, xylanases, mannanases). Pre-treatment methods can improve enzyme cellulose availability during enzymatic hydrolysis, which converts sugars into fermented ethanol [258].

Utilizing biofuels is an excellent alternative to diminish the use of petroleum oil, which leads to reducing carbon dioxide emissions, air pollution, and a safe environment.

The additional use of fungi to enhance environmental wellness is described by a new strategy named mycofiltration. Mycofiltration, or the process of treating contaminated water by passing it through a network of fungal mycelium, is one way that fungi are used in mycoremediation [259]. For example, a preliminary study by Taylor and their team proved the use of basidiomycete *Stropharia rugoso-annulata* as an adjuvant to improve synthetic stormwater *Escherichia coli* removal through wood chips [260].

#### 2.1.5. Research

In order to facilitate the study of particular biological phenomena, yeasts and filamentous fungi are used as research model organisms. Studies on these fungi provide relevant biological insights into other organisms, such as genetics, cell biology, meiosis, and pathogenesis. Yeasts and filamentous fungi are fascinating lower eukaryotes involved in understanding cellular processes. Their advantages are that they are easy to grow on inexpensive media and have easy access to molecular and classical genetics. The fact that fungi are more closely related to animals than plants underscores these organisms’ value as convenient models of human cells [261]. Fungi provide an excellent model for understanding the structure and function of chromatin in both actively transcribed regions (euchromatin) and transcriptionally silent regions (heterochromatin). *Saccharomyces* and *Aspergillus* are among the most prevalent fungi preferred by geneticists and molecular developmental biologists, but the first species used was *Neurospora*. Learning about epigenetic phenomena in other systems without the filamentous fungus *N. crassa* would have been challenging or impossible. *S. cerevisiae*, for instance, does not have the same characteristics in *Neurospora*, including DNA methylation and unique RNA interferences that act in mitotic and meiotic cells. Moreover, it contains an (RNAi)-based silencing system [262].

The initial usage of fungi for nanotechnology applications dates back to the early 2000s, making fungal nanotechnology a relatively new field of study. For example, palladium is a precious metal frequently used in catalysis, and in 2002, researchers at the University of California, Riverside, reported synthesizing palladium nanoparticles using a fungus called *N. crassa*. It has now been proven for the first time that fungi can operate as biological factories to create nanoparticles with distinct features. Since then, fungal nanotechnology has proliferated and has a wide range of potential uses, including the production of antibacterial agents, biosensors, and drug delivery systems. In addition, researchers are investigating methods to create various nanoparticles with varying shapes, sizes, and surface qualities utilizing several fungal species, including *Aspergillus*, *Fusarium*, and *Trichoderma* [263]. Apart from *S. cerevisiae*, many other yeast species, such as *Kluyveromyces* (*K. marxianus* and *K. lactis*); *Pichia* (*Pichia pastoris* renamed *Komagataella phaffii*, *Pichia anomala* renamed *Wickerhamomyces anomalus*); *Hansenula polymorpha* (renamed *Ogataea polymorpha*) and *Yarrowia lipolytica* were described as suitable research models, especially for biotechnological studies. Members of *K. marxianus* species are Crabtree-negative, can metabolize a broad spectrum of low-cost feedstocks such as whey and dairy industry wastes, and presents an exceptional ability to grow at elevated temperatures, thus being helpful in their use as a versatile host for a wide range of applications in the food, feed and pharmaceutical industries [264,265]. *Y. lipolytica* is considered a real industrial workhorse—the most extensively studied non-conventional yeast. Members of this species are strictly aerobic and are used to produce a variety of industrially important metabolites such as lipids, biosurfactants, and enzymes. Additionally, *Y. lipolytica* was used as a research model for dimorphism studies in yeasts and as a popular system for expressing heterologous proteins [266,267]. *K. phaffii* was recognized as an important host for the industrial production of heterologous proteins due to its possibility to run high-density fermentation associated with high secretory efficiency and its specific eukaryotic post-translational modifications [268].

### 2.2. Damaging Effects

#### 2.2.1. Etiological Agents of Diseases in Plants, Animals and Humans

The pathogenic potential of microorganisms, in general, is described as their ability to invade the host and produce toxic compounds that affect the well-functioning host. Although fungi are widely spread in the environment and co-exist with many organisms, in certain circumstances, fungi, like any other microorganisms, can harm their host. Fungi are characterized by high resilience in stressful conditions and a unique ability to adapt to different environments. Infectious fungi can spread through the air and water and be transmitted by different carriers such as animals, humans, or plants [18]. Regarding the interaction of fungi with plant organisms, more than 80% of the plants from our planet are symbiotic with fungi. However, some of the fungal strains can sometimes enter inside plants through damaged leaves and stomata, thus turning themselves into plant pathogens with a high impact on plant health. Plant fungal pathogens can be classified into biotrophs, hemibiotrophs, and necrotrophs, according to the mechanism involved in pathogenicity and the time required for complete host damage [269].

Necrotrophic fungi infect many hosts and can produce and secrete large hydrolytic enzymes that degrade plant cell walls. Thus, their pathogenic effect is rapid, causing the host’s death [270].

Both biotrophic and hemibiotrophic fungi require living plant tissue for their development, so their negative impact is much slower than necrotrophic fungi. Biotrophic fungi interact with the living host via special haustoria hyphae and can secrete specific molecules that suppress the plant’s immune system. The invasion of the plant organisms is assured by appressoria, which is involved in the attachment of the fungus to the substrate, allowing the cell wall penetration using mechanical force followed by affecting the plant’s normal metabolism by taking its nutrients [271]. Hemibiotrophic fungi combine both biotrophic and necrotrophic invasion mechanisms. First, these fungal pathogens invade the plant organism through mechanisms similar to those described for biotrophic fungi, followed by a necrotrophic phase that ends with the death of the affected plant [269,271]. Although tremendous progress has been made in recent decades to prevent and combat the fungal contamination of crops, devastating crop yield losses are still a significant problem. Therefore, many fungal species were described as phytopathogens associated with significant economic losses. Members of the *Botrytis* genus include pathogens of monocotyledons and dicotyledonous plants. Among the 22 species of *Botrytis*, *B. cinerea* is considered the most damaging, being able to infect numerous hosts [76,267]. The other 21 species have a narrow host range, especially infecting monocotyledonous plants. Gray mold is the most common disease caused by *B. cinerea*, affecting the mature or senescent tissue of the dicotyledonous host. Usually, the contamination occurs on the field, but severe damage is caused during storage in an improper condition of crops [272,273,274]. *B. cinerea* routes of infection vary according to the plant species and the environmental conditions. In general, *B. cinerea* conidia attach to the plant surface and germinate. Later it forms germ tubs that differentiate simple appressoria and infection cushions, structures involved in host penetration. Many models for the establishment of *B. cinerea* were presented. However, in general, after host invasion, the fungi produce specific molecules to suppress the death of host cells, which allows the fungi to accumulate enough biomass. After that, the fungus replaces autophagy-suppressing molecules with proteins that promote the plant’s ability to secrete enzymes involved in apoptosis, thus leading to the plant tissue’s death [275]. *Cladosporium fulvum* is responsible for tomato leaf mold, a common disease affecting production. The disease is associated with the exposure of tomato plants to high temperatures and humid environments, which promotes the growth of the pathogenic fungus. Usually, leaves are the main organs infected, and after contamination on the foliar surface appears, irregular chlorotic spots and the leaves edge becomes curly and wilted [276]. *C. fulvum* is a biotrophic fungus belonging to the Dothidiomycete class. Its infectious cycle starts with the germination of the conidia and the development of hypha, which enters the host through open stomata. Within two weeks, *C. fulvum* produces many conidiophores that block the stomata and cause leaf necrosis [273]. The *Magnaporthe grisea* species complex includes many fungal species responsible for causing disease in grass and sedge crops such as rice, wheat, barley, maize, oats, and finger millet. *Magnaporthe oryzae* occupies a central position among members of this group since it is a hemibiotrophic ascomycete known as the etiological agent of rice blast disease. This fungus mainly affects the rice plant’s aerial parts, leading to leaf, collar rot, and node blasts [277]. When invading the host, *M. oryzae* conidia develop melanized appressorium penetrating the rice cell wall through mechanical pressure. After that, the primary hyphae spread through plant cells and form a complex structure responsible for the secretion of effectors that suppress the plant’s defense responses. In time, the fungus changes its metabolism, secreting other toxins responsible for inducing the death of the plant tissue [273]. Another fungal species considered phytopathogenic is *Ustilago maydis*. Members of this species are also biotrophic plant pathogens responsible for causing corn smut disease. In this case, rapid proliferation is triggered after invading the host, which is associated with the development of tumors. Although this species has fewer damaging crops, *U. maydis* is considered a prime model organism for smut fungi characterized by a biphasic life cycle. The triggering of the pathogenic character is associated with the formation of diploids due to the mating of haploid cells that have contaminated the host plant [278]. *Mycosphaerella graminicola* (*Septoria tritici*) is responsible for septoria tritici blotch (STB)**,** the most important foliar disease of wheat, which is associated with necrotic lesions on leaves and stems [279]. Members of this species are well characterized regarding their pathogenic character. This pathogen is spread by wind, and its propagation is ensured by both sexual ascospores and asexual pycnidiospores [280].

As mentioned before, the pathogenicity of a microorganism is strongly influenced by the affected host. Therefore, when an organism is analyzed as a potential host for microbial infections, it must be taken into account that in terms of intracellular organization, there are some similarities between plant and animal cells (organelles with similar or standard functions, cytoskeleton elements a.s.o), but the differences between them are that they are significantly more numerous when discussing the cell wall or the cell membrane. More than that, in the case of multicellular organisms, the plant cellular immune systems are entirely different from those of animals. In these circumstances, plant and animal pathogens have a divergent evolution that allows them to adapt to the conditions encountered in the infected organism [245].

Despite their divergent evolutionary pathways, there are some pieces of evidence that phytopathogenic fungi can cause infections in humans or animals. A good example is supported by another *Alternaria* species, *Alternaria alternata*, which is a phytopathogenic fungi [281]. In addition, *Alternaria infectoria*, a fungal species responsible for causing severe blossom blight [282], might also be involved in causing phaehyphomycosis after renal transplant [283]. Other examples are the members of the *Cladosporium* genus, which were isolated both from infected plant and animal hosts [18,284,285,286].

Among the numerous amounts of different types of fungi, only a tiny percentage of these species are responsible for human infections or diseases. However, these pathogens cause various infections, starting from affecting internal organs to superficial infections of the skin and mucosal surfaces, allergies, and mycoses, and are more likely to arise in immunocompromised people with weakened immune systems [287,288]. Since the human body temperature is a significant barrier to fungal development, only a few fungi are potentially harmful organisms. Therefore, they are frequently seen outside the human body, such as ringworm or athlete’s foot [289].

The most common fungal infection is represented by fungal nail infection. The principal fungi responsible are fungi from the genera *Trichophyton* and *Microsporum*. This infection enters the nail bed and diffuses into the margins on the sides of the upper side of the nail, causing the nail to develop a white surface, discoloration, and thickening. In addition, this infection causes the skin around it to become scaly. Chlorazol Black E is used in microscopic and staining procedures to identify the onchomycosis. Chemical remedies or surgery are both options for treating nail infections. Terbinafine and itraconazole are just a few medications used to treat onchomycosis. However, they all have some adverse side effects. The typical infection is athlete’s foot, mostly in wet areas [290].

*Cryptococcus neoformans*, a fungal pathogen, can cause fatal fungal pneumonia and meningitis among immunocompromised patients. Naturally, it lives on waste and soil polluted with chicken, pigeon, and bat excrement. It penetrates the brain, extrapulmonary tissues, and the lungs. These infections most frequently result in infections of the lungs, skin, prostate, central nervous system, and eyes. If left untreated, it produces deadly cryptococcal meningoencephalitis. Antifungals such as flucytosine and intraventricular miconazole are used during treatment [288].

*A. fumigatus* is the fungus that causes aspergillosis. It is a saprophytic fungus that makes asexual spores from vegetative mycelium found in soil. The inhalation of *A. fumigatus* conidia results in lung infection. These strains can lead to invasive fungal infection in people with deficient immune systems. Patients with compromised immune systems can develop chronic pulmonary aspergillosis, one of the most prevalent invasive fungal diseases. Galactomannan antigen may be used to diagnose it. In addition, antifungal medications are used to treat aspergillosis. To treat aspergillosis, ergosterol, components of the fungal membrane, and 1,3 glucan are among the potential targets for antifungal compounds [288,291].

A healthy part of the human microbiome is *C. albicans. C. albicans*, however, transforms into a pathogen when the harmonious relationship between the organism and the host cells is perturbed. It then overgrows on skin and mucosal surfaces, invades host tissue, spreads to circulation, and colonizes solid organs. Patients with this illness have excruciating pain and agitation, especially immunocompromised patients. Oropharyngeal and esophageal candidiasis are both conditions, and the agent that causes them is *C. albicans* [292].

*R. oryzae* is a fungus that causes a medical condition known as mucormycosis. It is a member of the *Mucorales* order. Based on where the illness occurs, mucormycosis can be classified into five different groups: gastrointestinal, cutaneous, pulmonary, disseminated, and miscellaneous. Patients with neutropenia and dysfunctional phagocytes (caused by acidosis and hyperglycemia) are more likely to contract the illness. Mucormycosis etiology is associated with higher patient serum iron, such as cryptococcosis. Therefore, a quick and accurate diagnosis is crucial for illness therapy. Unfortunately, no PCR-based or serological assays are available for quick diagnosis. Therefore, treatment includes quick detection and surgical excision of the diseased tissue. Treatment options include quick diagnosis, surgical removal of the infected tissue to stop further invasion, and the use of antifungal medications such as amphotericin B deoxycholate and its lipid derivatives, azoles such as itraconazole, voriconazole, posaconazole, and ravuconazole, investigational triazoles, and echinocandins such as caspofungin [288,293].

#### 2.2.2. Mycotoxin Production

Mycotoxins are toxic secondary metabolites produced by numerous fungal species with a high negative impact on the health of humans and livestock, which can persist in food commodities after harvesting or processing [294]. Although in recent decades tremendous advances in understanding the biochemistry, genetics, and regulation of mycotoxin biosynthesis have been made, mycotoxin contamination of food products remains a problem far from being solved. These secondary metabolites are produced by toxigenic fungi belonging to *Aspergillus*, *Fusarium*, *Talaromyces*, and *Penicillium* genera. In food security and safety, their involvement in reducing the quality and quantity of food commodities requires intensive research [295]. Today, more than 300 mycotoxins of fungal origin are known, and their chemical structure varies from simple molecules with four carbon atoms to more complex ones. Although mycotoxins are secondary metabolites of different fungal strains, they do not intervene in fungal growth but rather act as a defensive mechanism against other organisms and as a strategy to maintain the oxidative status of the fungal cell [296]. In general, there are six types of mycotoxins considered most dangerous for human health: aflatoxins, ochratoxins, trichothecene, patulin, fumonisins, and zearalenone [297,298,299].

##### Mycotoxins Biosynthetic Pathways—Mechanisms and Genetic Background

**Figure 1:**
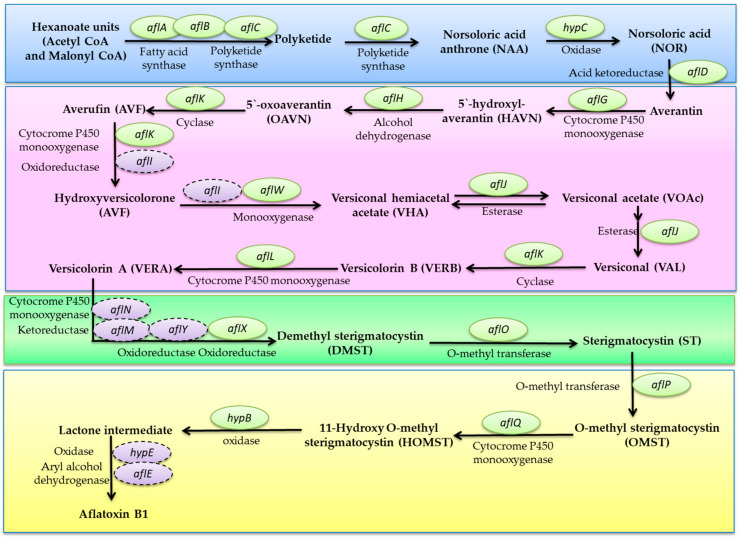
*Aspergillus* spp. aflatoxin B1 biosynthesis pathway and the genes involved (according to [305,312,314,317,318] (green circles with full border- genes with proven function; purple circles with dashed edges- putative genes).

Aflatoxins are produced mainly by *A. flavus* and *A. parasiticus* strains, found in soil, decaying vegetation, or grains, and less frequently by *Aspergillus bombycis*, *A. ochraceus*, *Aspergillus nomius*, and *Aspergillus pseudotamari* species. Depending on their fluorescence underneath UV light and relative chromatographic mobility, four main types of aflatoxins (B1, B2, G1, and G2) were described [297], but based on their toxicity, B1 aflatoxin is considered the most genotoxic [292,293]. Aflatoxin B1 (AFB1) biosynthesis is a complex process that involves at least 27 enzymatic reactions. Based on the intermediates formed during the entire cascade, the biosynthesis pathway of AFB1 can be divided into four main stages. The first stage is the conversion of acetate into norsolorinic acid. The cascade reaction starts with forming hexanoate units from acetyl-CoA and malonyl-CoA and their transformation into norsolorinic acid (NOR). The first reaction is catalyzed by two fatty acid synthases encoded by *aflA* (*fas-2*) and *aflB* (*fas-1*) genes. After that, the acetate derivatives are subjected to chain elongation catalyzed by a polyketide synthase encoded by *the aflC* (*pksA*) gene [300,301,302,303]. The resulting norsolorinic acid anthrone (NAA) is oxidized to norsolorinic acid (NOR) by the anthrone oxidase HypC encoded by *the hypC* (*hypB1*) gene [304] (Figure 1, blue color). The second stage of AFB1 synthesis is converting norsolorinic acid into versicolorin A through a series of 10 enzymatic reactions. First, the norsolorinic acid is transformed into averantin (AVN) by a ketoreductase encoded by the *aflD* (*nor-1*) gene. The averantin is hydroxylated in a reaction catalyzed by a P-450 monooxygenase encoded by *the aflG* (*avnA*) gene to form 5’-hydroxyaverantin (HAVN), which is subsequently converted to 5’-oxoaverantin (OAVN) through a reaction catalyzed by an alcohol dehydrogenase encoded by *aflH* (*adhA*) gene. A cyclase encoded by *aflK* (*vbs*) catalyzes the reaction of transforming 5’-oxoaverantin (OAVN) in averufin (AVF), which is further transformed to versiconal hemiacetal acetate (VHA) through two successive reactions catalyzed by monooxygenases (a P450 monooxygenase encoded by *aflV* (*cypX*) and a cytosolic monooxygenase encoded by *aflW (moxY*)), the intermediate compound being hydroxyversicolorone (HVN). In this process, an enzyme encoded by *the aflI* (*avfA*) gene also intervenes, which might be responsible for the reaction needed for the ring-closure step in the formation of hydroxyversicolorone (HVN) [301,303,305]. Versiconal hemiacetal acetate (VHA) is further transformed into versiconal (VAL) by an esterase encoded by *the aflJ* (*estA*) gene, and versiconal (VAL) is transformed into VERB through a reaction catalyzed by the cyclase encoded by *aflK* (*vbs*) [306]. Versicolorin B (VERB) is the critical branch point leading to forming AFB1/AFG1 or AFB2/AFG2. VERB contains both a tetrahydrobisfuran ring (such as AFB2/AFG2) and a dihydrobisfuran ring (such as AFB1/AFG1), so the conversion of versicolorin B (VERB) to versicolorin A (VERA) requires the desaturation of the bisfuran ring under the action of a P450 monooxygenase encoded by *aflL* (*verb*), whose activity is highly dependent of the cultural conditions [303,307] (Figure 1, pink color). The third stage of AFB1 biosynthesis is the conversion of versicolorin A into sterigmatoxystin through a series of three successive reactions catalyzed by different enzymes encoded by *aflM* (*ver-1*), *aflN* (*verA*), *aflY* (*hypA*), *aflX* (*ordB*) and *aflO* (*omtB*) genes. The intermediate compounds formed are not yet fully understood, but the final step of this stage is the conversion of demethylsterigmatoxystin (DMST) into sterigmatocystin (ST) through a reaction catalyzed by an O-methyltransferase [301,308,309] (Figure 1, green color). The last stage of AFB1 production is the conversion of sterigamtocystin into aflatoxin B2. The conversion of sterigamatocystin into O–methylsterigmatocystin (OMST) is catalyzed by an O-methyltransferase encoded by *the aflP* (*omtA*) gene, whose expression is highly influenced by the growth conditions [310,311]. The A-ring of O–methylsterigmatocystin (OMST) is oxidized, and an intermediate named 11-hydroxy-O-methylsterigmatocystin (HOMST) is formed. The reaction is catalyzed by a P450 monooxygenase encoded by *the aflQ* (*ordA*) gene. Moreover, 11-hydroxy-O-methylsterigmatocystin (HOMST) is further oxidized to a lactone intermediate by an oxidase encoded by *hypB* (*hypB2*), whose expression is also strongly influenced by the culturing conditions, and the final steps of aflatoxin B1 biosynthesis are catalyzed by different enzymes most probably encoded by *hypE* (*aflLa*) and *aflE* (*norA*) genes (Figure 1, yellow color) [301,312,313].

Many genes involved in aflatoxin production are organized as a cluster from chromosome 3 in the case of *A. flavus*. A similar cluster was also described for *A. parasiticus*, the second-most-critical fungal species that can produce large quantities of aflatoxins. Between the two of them, the homology of the clustered genes is 90–99%, and in terms of functionality, the main difference is that *A. flavus* strains are mainly producers of B types of aflatoxins while *A. parasiticus* strains produce both B and G types. Although many questions regarding the biosynthetic pathway of AFB1 have found their answer, many other problems remained unsolved in recent years. Apart from the genes that encode enzymes directly involved in AFB1 biosynthesis for other genes, such as *aflT* and *hypD* (*aflNa*), the function was not fully elucidated [301,314]. In the case of *A. flavus*, the aflatoxin pathway is regulated by a transcriptional factor encoded by *the aflR* gene. The Cys6Zn2 transcriptional factor binds to at least 17 genes from the aflatoxin genes cluster and acts as a positive regulator enhancing their transcription and, thus, aflatoxins production up to 50 times. Similar transcriptional factors were also characterized for *A. parasiticus* and *A. nidulans* strains [301,315]. Another possible transcriptional factor in the aflatoxin biosynthesis pathway is a protein encoded by *the aflS* gene. This factor might influence the expression of *aflC*, *aflD*, *aflM*, and *aflP* genes, but its exact mechanisms of action are not yet fully elucidated [301,305,316].

**Figure 2:**
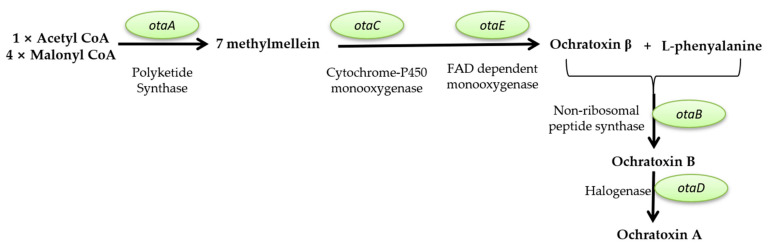
Ochratoxin A biosynthesis pathway and the genes involved (according to [318,319,325,326,327]) (green circles with full border- genes with proven function).

**Ochratoxins** are polyketide-derived secondary metabolites classified into three main types: A, B, and C. Chemically, ochratoxin A (OTA) contains a dihydrocoumarin moiety coupled to L-β-phenylalanine and is considered to be the most toxic [319]. Ochratoxin B (OTB) is the non-chlorinated form of ochratoxin A, while ochratoxin C (OTC) is an ethyl ester form of ochratoxin A [320]. OTA is produced mainly by *Aspergillus* (*A. ochraceus*, *Aspergillus carbonarius*, *A. niger*, *Aspergillus alliaceus*, *Aspergillus sclerotiorum*, *Aspergillus sulphureus*, *Aspergillus albertensis*, *Aspergillus auricomus*, *A. fumigatus*, *A. versicolor*, *Aspergillus pseudoelegans*, *Aspergillus roseoglobulosus*, *Aspergillus westerdijkiae*, *Aspergillus welwitshiae* and *A. wentii*) [319,321,322] and *Penicillium* (*Penicillium verrucosum*, *Penicillium nordicum*, *P. expansum* and less frequently *P. chrysogenum*) [319,323,324] fungal strains, and it is found in many types of food commodities such as cereals, dried fruits, nuts, and oilseeds. OTA biosynthetic pathway starts with 7-methylmellein production from acetyl-CoA and malonyl-CoA through a reaction catalyzed by the OtaA enzyme (encoded by PKS gene-*otaA*) which is a polyketide synthase. Then, 7-methylmellein is further oxidized to β-ochratoxin; the reaction is catalyzed by a cytochrome P450 monooxygenase named OtaC (encoded by *otaC*). Subsequently, β-ochratoxin is combined with L-β-phenylalanine by NRPS enzyme (*otaB*), resulting in an amide bond and OTB. The next step involves the chlorination of OTB by a halogenase named OtaD (*otaD*), thus resulting in the final compound OTA (Figure 2). 

The genes involved in OTA biosynthesis, in the case of *A. ochraceus*, *A. carbonarius*, *A. niger*, *A. steynii*, and *P. nordicum*, are grouped as a cluster formed by five genes, of which four are responsible for encoding enzymes (*otaA*, *otaB*, *otaC*, *otaD)*. One is responsible for the production of a transcription factor—*otaR1*. Additionally, there are two other genes relevant to the OTA biosynthetic pathway *otaE*, which encodes a flavin-adenine dinucleotide-dependent oxidoreductase and a second transcriptional factor (encoded by *otaR2*). Thus, the OTA biosynthetic pathway regulation is ensured by the otaR1 transcriptional factor, which controls the expression of *otaA*, *B*, *C*, and *D* genes, and the otaR2 factor responsible for modulating the expression of *otaA*, *B*, and *D* [318,325,326].

**Figure 3:**
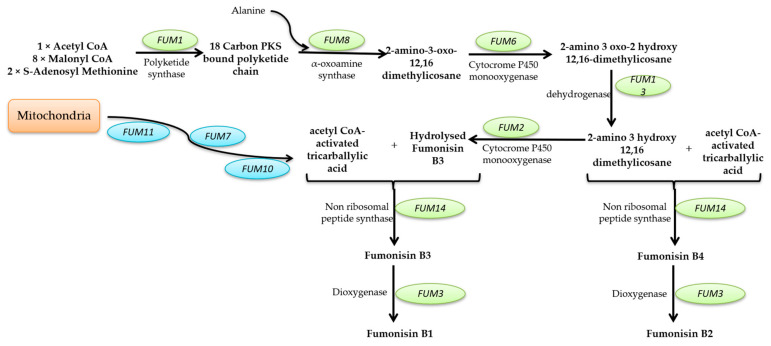
General aspects regarding fumonisins biosynthesis pathway and the genes involved (according to [318,328,330,336]) (green circles—genes with proven function; blue circles—gene-encoding enzymes involved in acetyl CoA tricarballylic acid transportation or processing *FUM11-*tricarboxyl transporter; *FUM7-*alcohol dehydrogenase; *FUM10-*Acyl CoA synthase).

Fumonisins are polyketides consisting of a 19- or 20-carbon backbone with hydroxyl, methyl, and tricarballylic acid moieties, produced mainly by *Fusarium verticillioides*, *Fusarium proliferatum*, and *Fusarium nygamai* fungi that contaminate maize and maize-derived products [297,328]. These mycotoxins are classified into four major groups: A, B, C, and P, but only B (fumonisins B1, B2, B3, and B4) and A (fumonisins A1 and A2) groups are considered extremely important for human health; most frequently they are encountered in the human food chain [329]. Among group B fumonisins, fumonisin B1 is the most abundant in contaminated foods, representing up to 80% of the total fumonisin percentage [330]. The precursors for fumonisins biosynthesis are acetyl-CoA, malonyl-CoA, and S-adenosyl methionine. One molecule of acetyl-CoA, eight molecules of malonyl-CoA, and two molecules of S-adenosyl methionine are used to obtain 18-carbon PKS-bound polyketide chain compounds through a reaction catalyzed by polyketide synthase (PKS) encoded by *FUM1.* The α-oxoamine synthase encoded by *FUM8* catalyzes the reactions of the decarboxylation of the amino acid and acyl-CoA thioester, and two carbon atoms of alanine are incorporated into the intermediate, forming a 22-carbon compound (2-amino-3-oxo-12,16-dimethyleicosane). An intermediate is formed through a reaction of hydroxylation at C14 and C15 catalyzed by a decarboxylase encoded by *the FUM6* gene. This intermediate is further reduced to 2-amino-3-hydroxy-12, 16-dimethylicosane by a reaction catalyzed by a short-chain dehydrogenase encoded by *FUM13* [331]. Subsequently, another hydroxylation at C10 occurs under the action of an enzyme encoded by *the FUM2* gene resulting in hydrolyzed B3 fumonisin. The final stage of fumonisins production involves another precursor represented by an acetyl CoA-activated tricarballylic acid. The presence of acetyl CoA-activated tricarballylic acid is assured by the action of other enzymes and transporters encoded by *FUM7*, *FUM10*, and *FUM11* [318]. FB3 and FB4 are formed by the esterification of acetyl CoA-activated tricarballylic acid at the C14 and C15 carbon atoms of hydrolyzed fumonisin B3 or 2-amino-3-hydroxy-12,16-dimethylicosane through reactions catalyzed by enzymes encoded by *FUM14* [332]. The hydroxylation of FB3 and FB4 under the action of the *FUM3* encoded enzyme leads to the formation of FB1 and FB2 [318,328,330] (Figure 3). From a genetic point of view, many genes related to fumonisins production were identified in *the F. verticillioides* genome. However, a cluster formed by 17 co-regulated genes is essential among them. The cluster contains both enzyme-encoding genes such as *FUM1*, *FUM3*, *FUM6*, *FUM8*, *FUM 10*, and *FUM13* [318], transporters such as *FUM11* [332], and DNA-binding transcription factors such as *FUM21* [333]. More than that, in the same cluster are included genes that might be responsible for fungal self-protection during fumonisins production, such as *FUM17* and *FUM18* [334,335].

**Figure 4:**
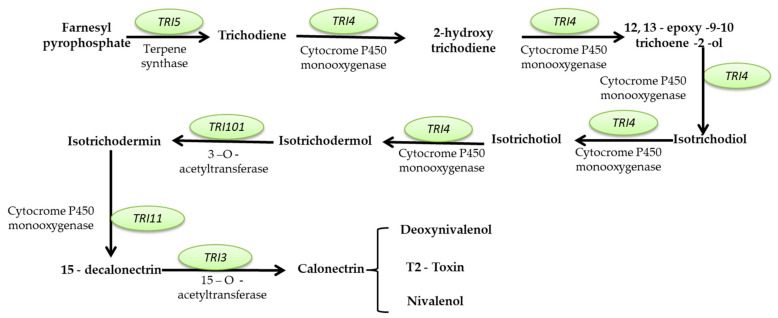
General aspects regarding trichothecenes biosynthesis pathway and the genes involved (according to [318,330,338,339]) (green circles—genes with proven function).

Trichothecenes represent the most diverse class of mycotoxins produced mainly by *Fusarium* species but also by *Myrothecium* and *Stachybotrys* strains. This group of mycotoxins contains more than 200 toxins, with molecular weights ranging from 200 to 500 Da. These compounds share a sesquiterpenoid structure. The presence or absence of macrocyclic esters or ester ether bridges between C-4 and C-15 and double bonds between C-9 and C10 with different side-chain substitutions ensures the differences between them. Many trichothecenes also contain 12,13-epoxyalkylene groups responsible for their cytotoxicity [337,338]. Trichothecenes are divided into four groups: type A containing T-2, HT-2, neosolaniol (ENNS) and diacetoxyscirpenol (DAS); type B that includes deoxynivalenol (DON) and its derivatives (3-acetyl deoxynivalenol and 15-acetyl deoxynivalenol), nivalenol (NIV) and Fusarenon-X; type C whose main representative compound is crotocin; and type D that includes roridin A, errucarin A and satratoxin H [337,339]. In the case of *Fusarium* species, the biosynthesis of trichothecenes starts with the cyclization of farnesyl pyrophosphate, resulting in trichodiene. The reaction is catalyzed by Tri5 synthase, encoded by *the TRI5* gene. Then, trichodiene undergoes a reaction of oxygenation catalyzed by a cytochrome P450 monooxygenase, encoded by *TRI4.* The oxygenation consists in adding four oxygen atoms at C-2, C-3, C-11, and C-12 positions, resulting in isotrichotriol. Through non-enzymatic isomerization and cyclization, isotrichotriol is transformed into isotrichodermol. The last compound is converted into isotrichodermin through a reaction catalyzed by an acetyltransferase encoded by *TRI101.* Then, a second hydroxyl group is added to C-15 by an enzyme encoded by *TRI11*, resulting in 15-decalonectrin. This compound is acetylated in the presence of the *TRI3* encoded enzyme, thus forming calonectrin [318,330,338,339] (Figure 4). The calonectrin compound can be considered an intermediate compound necessary for the biosynthesis of several types of trichothecenes, namely T-2 toxin, deoxynivalenol, and nivalenol.

**Figure 5:**
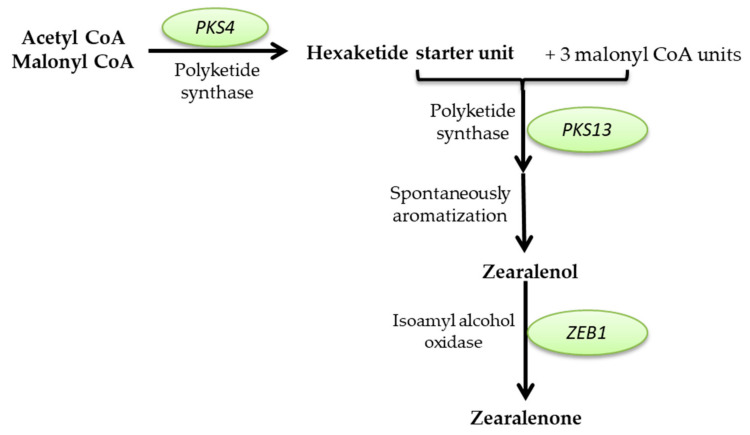
General aspects regarding zearalenone biosynthesis pathway and the genes involved (according to [318,340,341,342]) (green circles—genes with proven function).

Zearalenone (ZEN), previously known as F-2 toxin, is a resorcyclic acid lactone produced by several *Fusarium* species such as *Fusarium graminearum (Gibberella zeae)*, *Fusarium culmorum*, *Fusarium crookwellense (F. cerealis)*, *Fusarium semitectum* and *Fusarium equiseti*. Zearalenone and its derivatives (α-zearalenol (α-ZEL); β-zearalenol (β-ZEL); zearalanone-(ZAN); α-zearalanol (α-ZAL) and β-zearalanol (β-ZAL)) usually contaminate corn, barley, wheat, beer, and soybean [337]. Chemically, α-ZEL and β-ZEL differ from ZEN and ZAN by replacing the C6’ keto group with a hydroxyl group. The main difference between ZAL and ZEN and ZEN derivatives is ensured by the lack of a C-1’-C-2’ double bond [336,337,340]. In the case of F. gramniareum, four genes were described as being involved in ZEN biosynthesis: *PKS4*, *PKS13*, *ZEB1*, and *ZEB2.* Except for *ZEB2*, which encodes a transcriptional factor, the other three encode enzymes involved in biosynthesis. The first step of ZEN biosynthesis is condensing acetyl-CoA and malonyl-CoA molecules to form a hexaketide starter unit. The reaction is catalyzed by a polyketide synthase encoded by *PKS4* [341]. Then, the hexaketide undergoes transacylation and chain extension by adding three extra malonyl-CoA molecules. The reactions are catalyzed by a polyketide synthase encoded by the *PKS13* gene. After a spontaneous aromatization of the ZEN backbone, a compound with a macrolide ring structure and lactone bond named zearalenol is formed. This compound is further converted into zearalenone through a reaction catalyzed by an isoamyl alcohol oxidase, encoded by the *ZEB1* gene (Figure 5) [337,340].

**Figure 6:**
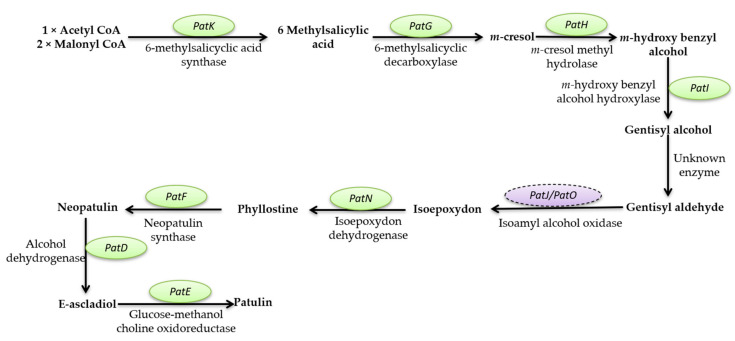
General aspects regarding patulin biosynthesis pathway and the genes involved (according to [345,346,349]) (green circles with full border- genes with proven function; purple circles with dashed edges- putative genes).

Patulin is a polyketide lactone frequently encountered in fruits and fruit products. Although initially investigated as a possible antimicrobial agent, today, patulin is considered a dangerous compound that affects human health when ingested [343]. Patulin is produced by many fungal strains belonging to *Penicillium* genera (*Penicillium carneum*, *Penicillium clavigerum*, *Penicillium concentricum*, *Penicillium coprobium*, *Penicillium dipodomyicola*, *P. expansum*, *Penicillium glandicola*, *Penicillium gladioli*, *P. griseofulvum*, *Penicillium marinum*, *Penicillium paneum*, *Penicillium sclerotigenum*, *Penicillium vulpinum*); *Aspergillus* genera (*A. clavatus*, *A. giganteus* and *Aspergillus longivesica*); *Paecilomyces saturnus*; and *Byssochlamys fulva* [344,345]. The biosynthetic pathway of patulin presents ten chemical reactions starting from one molecule of acetyl-CoA and three molecules of malonyl-CoA, which are condensed through a reaction catalyzed by a multifunctional enzyme-6-methylsalicyclic acid synthase (6MSAS) encoded by *PatK* gene to form 6-methylsalicylic acid (6MSA). The resulting compound is further transformed into *m-*cresol by 6MSA decarboxylase, and the methyl group of *m-*cresol is oxidized to form an aldehyde. After a hydroxylation reaction, it forms gentisyaldehyde. Gentisyaldehyde is converted into phyllostine through a reaction catalyzed by an isoamyl alcohol oxidase (*PatO)*, and the last compound is converted into neopatulin by neopatulin synthase (*PatF)*. The last step might involve the conversion of E-ascladiol into patulin via a ring-closing reaction mediated by a glucose-methanol-choline oxidoreductase encoded by *PatE*, which is similar to versicolorin B synthase, described in the AFB1 biosynthetic pathway. Although in recent years, many studies have made much progress in elucidating the patulin biosynthesis pathway, certain steps remain open to question. An example is represented by the reactions necessary for converting gentisylalcohol into gentisylaldehyde (Figure 6) [345,346,347]. More than that, the genes involved in patulin biosynthesis seem highly conserved. For *A. clavatus*, a cluster of 15 genes responsible for producing this toxin was described. In the same cluster are gene-encoding enzymes such as *PatK*, *PatG*, *PatI*, *PatO*, *PatD*, *PatF*, or *PatE*, specific regulatory factors, and transporters [345,348]. A similar cluster was described for *Penicillium* species, with the mention that, in their case, the order of the genes in the cluster is quite different. Despite that, the biosynthetic pathway is not influenced [346].

##### Health Implications of Mycotoxins Exposure

In 1962, when more than 100,000 turkeys from a poultry farm in London died after being fed with contaminated Brazilian groundnut meal, the “second mycotoxixology era” began. Later, it was proven that the incident was caused by secondary metabolites, today known as aflatoxins, produced by *A. flavus*. These fungal species contaminated the biomass used as feed on the farm [350]. More than 300 types of mycotoxins were described from this point forward, and many studies reported their negative impact on animal and human health. Only a few of the total number of mycotoxins described to date pose severe risks to public health, and most represent a real concern when they enter the human food supply chain (Table 3). Mycotoxins are classified according to their toxic activity into five main groups: Group 1, in which human carcinogens are included (e.g., aflatoxins); Group 2A, with probably carcinogenic mycotoxins for which no representative mycotoxins were described; Group 2B, with possible carcinogens (ochratoxins and fumonisins); Group 3, in which mycotoxins that are not yet considered human carcinogens (trichothecenes; zearalenones) are included **[351];** and Group 4, with mycotoxins that are probably not carcinogenic to humans [352]. Aflatoxin B1 is considered to have the highest carcinogenicity agent among mycotoxins since it can penetrate the cell membrane due to its liposoluble character. After entering the cell, it is rapidly metabolized to a highly reactive and unstable compound: aflatoxin-8,9- epoxide, which binds to DNA or proteins. The mutagenic effect of aflatoxin B1 consists of GC to TA transversions, directly affecting the function of the P53 gene, which encodes a tumor suppressor protein that inhibits the development of tumors [353,354]. In the case of ochratoxin A, the second dangerous mycotoxin, its carcinogenic effect is mainly based on the fact that this compound disturbs cellular physiology by disrupting the phenylalanine metabolism or by inhibiting the mitochondrial ATP production [353]. Although included in Group 2B of mycotoxins, fumonisins disrupt the sphingolipid metabolism and inhibit protein and DNA synthesis [353,355]. Mycotoxins present a real concern to public health because these compounds are widely spread into the world’s food supply [296]. Mycotoxigenic fungi are frequently encountered in all agricultural regions. Because of that, more than 20% of crops harvested worldwide are contaminated with mycotoxins (even those from the field) every year, followed by an increase in their concentration during storage [296,349].

#### 2.2.3. Deteriogenic Agents

Cultural heritage, known as a connection between the past and the present, requires specific safeguarding efforts because it brings together both tangible cultural heritage [significant elements that support the history and culture of a country, i.e., monuments (of different substrate types), books, art mills, paintings, photographs, parchments, mosques, sanctuaries, monasteries, churches, chapels, bridges of wooden, stone, museums] and intangible cultural heritage (traditional music, folklore, language, traditional practices, customs, customs, crafts, crafts, etc.) [386].

The biodeterioration of cultural heritage objects, involving aesthetic, architectural, and economic damages, is a big challenge for conserving cultural heritage materials and community safety. The available methods for preventing biodeterioration are most often effective when removing deteriogenic microbial biofilms but not when preventing the recontamination of heritage objects. In certain situations, the repeated use of current decontamination methods leads to complex alterations of heritage objects. The biodeterioration of heritage objects is currently a significant problem worldwide. Modern conservation strategies underline the need to know and understand the structure and function of complex microbial communities, which represents one of the leading causes of the biodegradation of the objects included in the cultural heritage. To achieve adequate conservation actions, it is necessary to identify community species of microorganisms and their activity on a substrate. Attempts by researchers to elucidate biogeochemical mechanisms of the biodeterioration process were perfected by introducing next-generation techniques and whole-genome sequencing. Then, the first step is represented by identifying the microorganisms responsible for the process that means fungi are resistant to drying out, and they benefit from spore diffusion as a transport mechanism. Fungi also produce a wide range of organic acids and provide mineral nutrients to other microorganisms, especially phototrophs [387].

##### The Most Frequently Encountered Cultural Heritage Objects and Their Deterioration by Fungi

Stone Objects

Stones are the primary structural components of ancient structures, starting from numerous types of buildings, monuments, statues, tombs, and weather conditions that have influenced their quality. Anthropogenic and natural forces mostly cause stone modification and decay because it is an extreme environment for biotic factors. The enounced forces cause various physical, chemical, and biological degradation. The lack of nutrients in their composition, water, and intense microclimate oscillation makes the stone objects an unfavorable surface for microorganism colonization. Although, if the conditions are convenient, cyanobacteria, algae, and lichens are considered the primary colonizers, whereas hemolithotrophic and hemoorganotrophic bacteria and fungi have been described as the secondary colonizers [388,389].

Fungi may be the most significant deteriogenic species on exposed building stones due to their great erosiveness [86,390]. Fungi may enter the stone depending on the material’s physical characteristics. Black fungi are the principal culprits behind the process known as bio-pitting, which results in fissures up to 2 cm in diameter and depth in stone. Although it has been seen on old glass, bio-pitting is most common on marble and limestone [391]. The most widespread stone-degrading fungi genera are presented in Table 4.

Stone may become damaged by fungi through chemical and physical processes. Chemical mechanisms include the release of acidic metabolites and pigments and the oxidation of cations that produce minerals. In contrast, physical mechanisms include hyphal penetration of the rock surface, which results in its fragmentation. Fungi are thought to be the most powerful creatures in nature that break down rocks and minerals, even though numerous microorganisms can produce acids. The creation of numerous secondary mycogenic minerals is caused by synthesizing various acidic metabolites, which cause the mineral substrate to dissolve through biocorrosion [388].

Wooden Objects

The majority of cultural heritage objects were manufactured using prime material wood. In accordance with UNESCO, wooden cultural heritage items can be divided into three categories: moveable, such as furniture, frames, sculptures, and musical instruments; immovable, for example, buildings such as windmills, monasteries, churches, temples, and bridges; and underwater, such as shipwrecks, foundation piles, wooden cargo or contents that were partially or entirely underwater, periodically or continuously, for at least 100 years [392].

Almost each wooden cultural heritage object is constantly undergoing chemical changes, and an expected action that may appear is degradation over time. Depending on the wood species that may be particularly robust or the exposure circumstances that may be moderate or non-aggressive, the degradation of wood items could be quite slow [393]. However, many of these objects are deteriorating and vanishing due to neglect and unintentional mistreatment. Because of the agents of deterioration, the wood species, and the climatic conditions, the level of wooden item degradation ranges from a nearly everyday look to being disintegrated and significantly altered. Among the factors of deterioration, we can name biotic factors such as the action of fungi, bacteria, or insects, and abiotic factors, such as humidity, temperature, and weathering [86,392].

Although all types of microorganisms can contribute to the destruction of wood cultural items, fungi have the most ability to degrade this particular category. The mechanisms of biodeterioration can imply the growth of fungus on the surface or between internal components, the generation of extracellular enzymes, and the structural modification of fundamental biopolymers, which finally leads to alterations of the item evident to the naked eye [388].

Most of the time, filamentous fungi can be recognized easily due to their macroscopic aspects of culture. They have fast growth on the surface of the substrates, where the conidia develop rapidly. Due to the specific color of the conidia, wood colonized by a multitude of mold species will present a multicolored image on its surface, such as black, given by the presence of *A. niger*, or green, given by *Trichoderma* spp., *Penicillium* spp. The species of *Trichoderma* spp. occur most frequently on beech roots, and beech stems that are stored are frequently colonized by *Bispora antennata*, causing black spots. Molds develop on fresh cuttings after felling trees and usually on those improperly stored and undergoing not-tolerable conditions. The hyphae penetrate the wood only a few millimeters and develop on the parenchyma cells. Moldy wood loses all its value. Using it for decorative purposes is impossible; it is not suitable because color spots cannot be removed mechanically but can only be hidden by paint. Infected wood is not suitable to be used for hygienic reasons; for example, it is not suitable to be used for packaging [394]. Untreated, the molds that appear on the surface of the wood are dangerous for its durability over time. Fungi will degrade the material, changing its composition and losing its original characteristics.

Three types of fungi alterations that destroy wood are known as white rot, brown rot, and soft rot. The most frequently encountered genera are included in Table 4.

Wood degradation can be evaluated by X-rays and computed tomography. This is a non-destructive alternative that allows the determination of the material’s structural state. This is crucial for cultural preservation because it enables an accurate definition of the state of deterioration, which helps develop a conservation strategy to stop the loss of heritage assets. For example, Alfieri et al. analyzed the wood alteration of locomotive wood slats by a white rot fungus, *Phellinus chaquensis*. They used X-rays and computed tomography to observe the area filled by mycelium and basidiomata and later processed the images. Therefore, they could observe the area affected by wood decay fungi [395].

Paintings

Oil painting on canvas has become one of the most significant art expressions, resulting in remarkable works with significant historical and cultural worth. In addition, oil paintings on canvas may offer the most remarkable diversity of microhabitats and nutrients utilized by a wide range of microbial species compared to other artworks [86,396,397].

The chemical composition of canvas paintings is diverse and includes organic and inorganic elements. Textile fibers are frequently used as the foundation material in oil canvas paintings. Glues, gelatin, casein, egg yolk, flour, rubber, oil, or resin are frequently used in the ground, pictorial, and protective layers [396]. These substances serve as nutritive substrates for different microorganisms that can colonize canvas paintings and are thus vulnerable to deterioration. The pigments often contain inorganic chemicals, frequently heavy metal compounds, which may have harmful effects and prevent the growth of some fungi and bacteria [398].

Another substrate that can be deteriorated by fungal activity is represented by wall paintings. As with the oil paintings, the wall paintings are also a substrate rich in nutrients (egg whites, milk, oils, casein, etc.) that suffice as a microhabitat for fungal growth. The most frequently reported fungi on canvas oil and wall paintings are included in Table 4.

Textiles

Textile biodeterioration is a prevalent issue affecting various materials with various chemical compositions broken down by various microorganisms in various environmental settings. Of course, the deterioration method differs for each type of product, and the care of several circumstances, such as usage, storage, cleaning, and exposure to climatic conditions, can prevent it. However, as it offers a mechanism to eliminate waste textiles in the environment, the microbiological deterioration of textiles is not necessarily bad. Therefore, when considering final usage and disposal, adding chemical protectants to textiles is only sometimes required. Due to the organic composition of most textile products, microorganisms deteriorate quickly because they are a source of nutrients. Therefore, synthetic fibers are less susceptible to deteriogens than fibers made from natural source materials. Weaves and other textile-based products show signs of biodeterioration as exterior changes, most frequently discoloration and related disagreeable odors. The chemical alterations brought on by the development of microorganisms cause diminished weave strength and eventually result in a partial or complete material breakdown [399]. The most widespread deteriogenic fungi are included in Table 4.

Paper and Paper-Based Materials

Paper-based materials are typically stored in libraries, archives, and museums. These include books, essays, antique maps, photos, and more. In addition, paper is the world’s most significant material for recording and preserving historical events, primarily created through the mechanical and chemical processing of cellulose fibers derived from wood. Cellulolysis, the enzymatic hydrolysis of cellulose polymer into glucose units, is a process by which fungal microorganisms may break down cellulose fibers. In this regard, isolating the fungus capable of producing cellulolytic enzymes from paper, mainly from ancient books or papers stored in libraries, archives, and museum depots, is common practice. Members of the genera *Chaetomium*, *Penicillium*, *Aspergillus*, *Eurotium*, *Trichoderma*, and so on are among the most common genera responsible for paper deterioration (Table 4) [86].

Parchments

Because ancient parchment-based writings were a crucial tool for human communication, they are of immeasurable historical importance to our society. Before the Middle Ages, they were the primary writing materials, but since the invention of paper, parchment has mainly been employed for noble reasons [400].

Collagen, the primary constituent of treated animal skin, is used to make parchment. Numerous microorganisms can degrade collagen, which they use as a source of energy and carbon. In parchment deterioration and that of other archival materials, fungi play a crucial role. As a result of this biodeterioration, parchment begins to lose some of its original qualities. For example, it becomes distorted, and there is a chance that white films, fading lettering, and spots may appear. The biodeterioration of parchment is frequently caused by the action of fungi that have extracellular enzymes that enable them to metabolize protein, generating various spots of different colors (brown, black, or reddish). In addition to this chemical activity, fungal hyphae can break down the parchment’s fiber structures and cause mechanical harm to the document. Table 4 includes the most common fungal deteriogenes genera that can colonize and degrade parchments.

**Table 4:** Deterioration of different cultural heritage substrate types by fungi.

Deteriogenic Agents/Group	Genus	Substrate	Alteration Types	References
Ascomycota Phyllum	*Acrodictys* spp.	stone artifacts		[401]
Dothideomycetes class	*Aureobasidium* spp.		black patina, black spots, biofilm formation, discolorations, stone erosion, and disintegration	[402,403,404]
	*Capnobotryella* spp.		black spots, crater shaped lesions, superficial deposit, and biofilm formation	[403,405]
	*Coniosporium* spp.		black patina, black spots, pitting, exfoliation, superficial deposit, and biofilm formation	[403,406]
	*Phoma* spp.		black spots, black patinas, exfoliation, pitting, superficial deposit, biofilm formation	[404,407,408]
	*Alternaria* spp.		black spots, black patina, biofilm formation, black crusts	
	*Cladosporium* spp.		black spots, patinas, pitting, biofilm formation, erosion, discoloration, disintegration	
	*Epicoccum* spp.		black spots, black patinas, superficial deposit, biofilm formation with salt efflorescence	
Eurotiomycetes class	*Exophiala* spp.		black patina, black spots, detachment of discolorations, visible damage	[389,406]
	*Knuffia* spp.	stone	black spots, patinas, pitting, discolorations, visible damage	[389,405,406,409]
	*Lithophyla* spp.		black patina, black spots	[245,402]
Dothideomycetes class	*Chaetomium* spp., *Aureobasidium* spp., *Epicoccum* spp., *Cladosporium* spp.	wall paintings	brown discolorations, degrade protein binders of the painted layer, which results in the lifting and separation of the painted layer from the support	[86,410,411]
Eurotiomycetes class	*Penicillium* spp., *Aspergillus* spp.		primary fresco deteriogens, damage of wall paintings due to its intensely sporulation degree	[86,412]
Zygomycetes class	*Mucor* spp., *Rhizopus* spp., *Actinomucor* spp.		surface contaminants	[413,414]
Basidiomycetes class	*Coprinus* spp.		contamination	[413]
Dothideomycetes and Eurotiomycetes classes	*A. alternata*, *A. flavus*, *A. niger*, *A. versicolor*, *Aureobasidium pullulans*, *Chaetomium globosum*, *C. cladosporoides*, *Eurotium chevalieri*, and *P. chrysogenum*	canvas oil paintings	the detachment of the paint layer from the support, the loss of material due to the excretion of metabolites, esthetic changes of materials, biofilm formation, chromatic alteration of the painted surfaces and detachment of the support	[89,393,410]
Zygomycetes class	*Cunninghamella* spp., *Mucor* spp., *Rhizopus* spp. *Phycomyces* spp.		dust deposits	[398]
Basidiomycetes class	*Puccinia* spp.		contaminants	[415]
Basidiomycetes class	*Bjerkandera* spp., *Donkioporia* spp., *Fomes* spp., *Irpex* spp., *Phanerochaete* spp., *Pholiota* spp., *Pleurotus* spp., *Trametes* spp.	wooden	white rot fungi complete depolymerization and degradation of lignin, cellulose, and hemicellulose components	[86,416]
Eurotiomycetes class Sordariomycetes class	*Aspergillus* spp., *Fusarium* spp.,			[86]
Basidiomycetes class	*Antrodia* spp., *Coniophora* spp., *Coriolellus* spp., *Gloeophyllum* spp., *Paxillus* spp., *Poria* spp., *Postia* spp., *Serpula (Merulius) lacrymans*		brown rot fungi cellulose and hemicellulose decomposition and lignin degradation is restricted to methoxyl group demethylation	[86,417]
Dothideomycetes class	*Alternaria* spp., *Stemphylium* spp.,		soft rot fungi cellulose and hemicellulose decomposition and lignin degradation is restricted to methoxyl group demethylation, leading to discoloration and cracking pattern	[86,392,417]
Sordariomycetes class	*Chaetomium* spp., *Daldinia* spp., *Humicola* spp., *Xylaria* spp.			
Eurotiomycetes Sordariomycetes and Dothideomycetes classes	*Penicillium* spp., *Aspergillus* spp., *Eurotium* spp., *Myxotrichum* spp. *Trichoderma* spp., *Chaetomium* spp., *Acremonium* spp., *Paecilomyces* spp., *Stachybotrys* spp., *Myrothecium* spp., *Cladosporium* spp., *Bipolaris* spp., *Aureobasidium* spp., *Alternaria* spp., *Epicoccum* spp.	paper and paper-based materials	pigments and organic acid production, brown to red spots (foxing)	[86,418,419,420,421,422]
Basidiomycetes classTritirachiomycetes classes	*Bjerkandera* spp., *Tritirachium* spp.			
Zygomycetes class	*Rhizopus arrhyzus*			
Eurotiomycetes and Sordariomycetes classes	*Aspergillus* spp., *Penicillium* spp., *Microsporum* spp., *Trichophyton* spp., *Chaetomium* spp., *Fusarium* spp.,	textiles	wool fibers degradation	[86,423]
Zygomycetes class	*Rhizopus* spp.		wool fibers degradation	[423]
Eurotiomycetes class	*Chaetomium globosum*		silk deterioration, causing cracks and gaps in fibroin fibers	[424]
Dothideomycetes and Eurotiomycetes classes	*Alternaria* spp., *Aureobasidium* spp., *Cladosporium* spp., *Epicoccum* spp., *Penicillium* spp.	parchment		[86,425,426]
Ascomycota phyllum	*Diploospora rosea*		the detachment of large parts of the artwork’s preparative layer and the overlying illumination.	[427]

## 3. Conclusions

Fungi have been used as a cell factory to produce enzymes and small molecule compounds for almost a century. The biomass produced during these production processes has generally been considered a waste stream. This inconvenience may change in the future since fungal biomass is now being explored as the basis of sustainable biomaterials. In agriculture, the presented applications have the potential to improve crop yield, reduce the use of synthetic fertilizers and pesticides, avoid the use of toxic compounds, and promote sustainable agriculture practices. Therefore, further attention must be paid to uncovering the biomolecules from fungi for agriculture and pharmaceutical applications through studying metagenomics, genomics, and proteomics.

Humans are endowed by evolution with robust defenses against invasive fungal diseases, successfully treating many of them. However, we are still vulnerable to invasive fungal infections. People suffering from opportunistic and primary invasive fungal infections urgently need resources and research efforts to bring them new diagnostics and treatments regardless of commercial potential. Enormous work over the past three decades has opened vast new views in fungal biology; we can expand upon them to fulfill the promises of modern medical advances.

## Figures and Tables

**Table 1:** Antimicrobial agents produced by filamentous fungi.

Antimicrobial Agents	Producer	Active Against	Mode of Action	References
Non-ribosomal peptides				
Mycophenolic acid	*Penicillium brevicompactum*	several species	antibacterial, antifungal, antiviral, antitumor, antipsoriasis and immunosuppressive, anti-angiogenic activities	[26]
Penicillin	*Penicillium notatum*	*Staphylococcus aureus*		[27]
Penicillin G	*P. rubens*	*Streptococcus*, *Staphylococcus*, *Enterococcus*, *Clostrodium* and *Treponema* spp.	inhibit the peptidoglycan synthesis	[28,29]
Cephalosporin C	*A. chrysogenum*	broad spectrum antibiotic		[2]
Ribosomal peptides				
Amatoxin Phallotoxin	*Amanita* spp.	anticancer drugs	RNA polymerase II inhibitors	[30]
Ustilotoxin	*Ustilaginoidea virens*	cytotoxicity against different anticancer cell lines	anti-mitotic activity	[31]
Depsipeptides Beauvericin (A–H) Beauveriolide	*Fusarium*, *Alternaria*, *Calonectria, Cochliobolus*, *Cordyceps cardinalis*, *Ophiocordyceps communis* *Cordyceps militaris*	antimicrobial and insecticidal activity anti-aging activity against *S. cerevisiae*		[32,33,34,35]
Piperazines				
Roquefortine C	*Penicillium roqueforti*	acute toxicity in mice and dogs		[36,37]
Gliotoxin	*Aspergillus fumigatus*	antifungal activity against *Candida albicans* and *Aspergillus* spp.	inhibit the activation of lymphocyte B and T	[38,39]
Polyketides				
Griseofulvin	*Penicillium griseofulvum*	dermatophytes fungal infections in humans and animals;non-fungal inflammatory diseases; cardiovascular, antitumor and antiviral applications;	inhibit fungal cell mitosis and nuclear acid synthesis	[40]
Patulin	*Aspergillus clavatus*	mycotoxin, fungistatic activity against *Rhizoctonia solani*, *S. cerevisiae*, *Didymella bryoniae*, *Botrytis cinerea*, *Fusarium oxysporum*, *clerotium rolfsii*, *Pithium ultinum*	destabilization of of the plasma membrane integrity, blockage in rRNA, tRNA, and mRNA synthesis	[41,42]
Strobilurins	*Strobilurus tenacellus*, *Oudemansiella mucida*	antifungal activity	inhibit the transfer of electrons between complexes II and III of the electron transport chain in the mitochondria, resulting in impaired cell respiration and ATP synthesis	[43]
Uredinorubellin derivatives	*Torrubiella* spp.	antibacterial activity against *S. aureus* strains		[34]
Rubellins antraquinones	*Ramularia collo-cygni*	antiproliferative, cytotoxic, aggregation inhibitory and antimicrobial activity against *B. subtilis*, *S. aureus*, *S. aureus MRSA*, *Enterococcus faecalis* clinical and reference strains	phytotoxic activity	[44]
Viriditoxin	*Penicillium radicum*	antimicrobial activity against *S. aureus MRSA*	inhibiting FtsZ, the bacterial tubulin	[45]
Lindgomycin	Lindgomycetaceae family	antimicrobial (against Gram-positive and *C. albicans* strains) and antiviral activity		[24]
Lipopeptides				
Echinocandin B	*Aspergilllus nidulans*		inhibiting β (1,3)-glucan synthase	[46]
Pneumocandin B_0_	*Glarea lozoyensis*	antifungal activity against *C. albicans* and *Pneumocystis carinii*	inhibiting β (1,3)-glucan synthase	
Caspofungin	*G. lozoyensis*		blocking cell wall biosynthesis by inhibiting β (1,3)-D-glucan synthase	[47]
Micafungin	*Coleophoma empetri*			
Anidulafungin	*A. nidulans*			
Mulundocandin	*Aspergillus sydowii*	*Aspergillus niger*, *C. albicans*, *Candida non-albicans*		[48]
Anidulafungin	*A. nidulans*	*Candida parapsilosis*, *Candida guilliermondii* *Aspergillus* spp. *Fusarium* spp.		
Rezafungin		*Candida* spp., *Aspergillus* spp., *Pneumocystis murina*		[48,49]
Cryptocandin	*Cryptosporiopsis quercina*	antifungal activity against *Tricophyton rubrum*, *Sclerotinia sclerotiorum*, *Botrytis cinerea*		[50,51]
Lipodepsipeptide				
Aureobasidin A	*Aureobasidium pullulans*	fungicidal activity against *Candida* spp., *C. neoformans*, *Blastomyces dermatitidis*, and *Histoplasma capsulatum*	noncompetitive inhibition of the inositol phosphorylceramide synthase	[52,53]
Nucleosidic peptide				
Arthrichitin FR-90403	*Arthrinium phaeospermum and Kernia* spp.	*C. albicans*	chitin synthase inhibitors	[54]
Other peptides				
Aspergillomarasmine	*Aspergillus versicolor*	Gram-negative rods	inhibit the NDM-1 and VIM-2 metallo-β-lactamases	[55]
Cyclosporin A	*Tolypocladium nivenum*		immunosuppressive, anti- coronaviruses activity	[56]
Peptaibols	*Trichoderma reesei*	antimicrobial activity against *Alternaria alternata*, *Phoma cucurbitaceum*, *Fusarium* spp., *A. fumigatus*		[57]
Plectasin	*Pseudoplectania nigrella*	*Streptococcus pneumoniae*		[58]
Leucinostatin A	*Purpureocillium lilacinum*	antifungal activity against *Candida* spp. (including *C. albicans*, *Candida krusei*, *Candida tropicalis*, and *C. guilliermondii*); antitrypanosomal and antitumoral activities		[59,60,61]
Terpene derivated metabolites				
Enfumafungin Ergokonin	*Hormonema* spp. *Trichoderma* spp.	antimicrobial activity against *Bacillus subtilis*, *Cryptococcus neoformans*, *C. albicans*, *A. fumigatus*	glucan synthesis inhibitors	[62,63]
Antifungal metabolites				
Parnafungin	*Fusarium lavarum*	inhibits inhibit mRNA polyadenylation in *Candida albicans* and pathogenic fungi		[64,65]
Other pharmaceutical agents				
Lovastatin	*Aspergillus terreus*		hypercholesterolemia treatment	[2]
Mevastatin	*Penicillium citrinum*			[66]
Pravastatin	*Penicillium chrysogenum*			[67]
Other bioactive compounds				
Clavatol	*A. clavatus*, *Aspergillus clavatonanicus*	fungistatic activiy against *C. albicans*, *A. niger*, *F. oxysporum*, *Rhizoctonia solani*, *Pythium ultimum*, *Didymella bryoniae*, *B. cinerea*		[68]
Pyranonigrins A, B, C, D, E, S	*A. niger*			[69]
Pyranonigrins A and F	*Penicillium brocae*	Antimicrobial activity against Gram-positive and Gram-negative strains		[25]

**Table 3:** Fungal mycotoxins and their effect on human/animal health [295,296].

Mycotoxins		Source	Effect on Human/Animal Health	References
Aflatoxins	B1 B2 G1 G2	Maize, peanuts, copra, corn, coffee beans, rice, sorghum, soybean	Cause hepatic and extrahepatic carcinogenesis by inducing DNA single-strain breaks; Direct contact of AFB1 with skin can result in tumor formation, hair loss, erythema and ulcer; Decrease the proliferation of gastrointestinal epithelial cells; AFB1 is associated with genotoxicity in isolated epithelial cells of jejunum; In high doses, suppresses the alternative pathway of complement activation; Inhalation of AFB1 causes primary lung cancer; It inhibits the production and function of natural killer cells; Repeated exposure of mice to AFB1 causes the decline of CD3 and CD8 T cells in intestinal mucosa.	[353,356,357,358]
Ochratoxins	A	barley, wheat, coffee beans, citrus, grape, beer, fruits, soybean, cereals; dried fruits; breast milk of exposed mothers; smoked and salted dried fish; cheese	Causes porcine and poultry nephropathy; damages the integrity of renal epithelial cells; Long-term exposure is associated with impairment of renal function, which leads to enzymuria, polyuria; red tongue, thirst, bitter taste; Induces the increase in reactive oxygen species in rat proximal tubule cells, leading to the depletion of intracellular gluthatione and cell death; Exhibits a neurotoxicity effect in human astrocytes, acting as an anti-proliferation agent and mediating mitochondria-dependent apoptosis.	[359,360,361,362,363]
Fumonisins		Maize; rice, wheat, sorghum; barley, oats	Affect newborns neural tube development; cause brain lesion in horses and pulmonary edema in pigs; Disrupt the myelin synthesis process, causing leukoencephalomalacia in horses; Alter the cytokine profile of different organs and cell types, mediating the increased expression of TNF-α and interleukin-1β in mouse liver and kidney.	[337,364,365,366]
Zearalenone		Maize; wheat; barley; oats; grains; animal feed	Affect the reproductive system of laboratory animals, determining the appearance of changes in reproductive tract, reduced fertility, and increased embryo-lethal resorption, and affecting the progesterone and estradiol serum level; Induce liver lesions and alter the hepatic function of rabbits and rats; Stimulate the growth of human breast cancer cells.	[367,368,369,370,371]
Patulin		Fruits; fruit juices, cheese, wheat	Induces erythrocyte death (eryptosis) by stimulating the entry of Ca^2+^ inside the cells; Repeated exposure of rats to sub-acute administration of patulin causes neurotoxicity (tremors, convulsion) and ATPase inhibition with direct consequences in lipid metabolism; Long-term exposure leads to decreasing sperm count.	[372,373,374]
Trichothecenes	Deoxynivalenol	Maize; wheat; barley; oats; grains; animal feed	Vomiting, digestive disorders, oxidative damage, reproductive toxicity; Inhibits protein and nucleic acid synthesis through direct binding of ribosomal peptide transferase active sites and activation of cell kinases; Triggers MAPK mediated up-regulation of pro-inflammatory cytokine expression and apoptosis; Exhibit strong embryo toxicity (decreasing fetal body weight, crown-rump length and vertebral ossification).	[337,375,376,377,378]
	Nivalenol		Increase levels of MAPKs and phosphatase; Causes nausea, diarrhea, and vomiting; Increases IgA concentration and its accumulation in the glomerular mesangium; Inhibits the proliferation of human mitogen-stimulated lymphocytes, thus exerting immunosuppressive effects.	[337,379,380]
	T-2 toxin and HT-2 toxin	Maize, oat, barley, wheat, rice, soybean	Acute poisoning symptoms such as: nausea, abdominal pain, diarrhea, bloody stools, weight loss and decreased immunity; Affect cell cycle, causing chondrocytes, astrocytes, hepatocytes and epidermal basal cell apoptosis; TReduce antibody formation and alter leukocytes counts; Affect the innate immune response by decreasing the activity of blood alkaline phosphatase.	[337,381,382,383,384,385]

## Data Availability

Not applicable.
